# Life history traits of the target pest and transmission routes of the biocide are critical for the success of the boosted Sterile Insect Technique

**DOI:** 10.1016/j.cris.2024.100101

**Published:** 2024-11-12

**Authors:** Fanny Herbillon, Esther Gnilane Diouf, Thierry Brévault, Marion Haramboure, Simon Fellous, Cyril Piou

**Affiliations:** a CIRAD, UMR CBGP, F-34398 Montpellier, France; b CBGP, Univ Montpellier, CIRAD, INRAE, Institut Agro, IRD, Montpellier, France; c ENSA, Université de Thiès, Thiès, Sénégal; d CIRAD, UPR AIDA, BIOPASS, Centre de recherche ISRA-IRD, Dakar, Sénégal; e CIRAD, UPR AIDA, F-34398 Montpellier, France; f AIDA, Univ Montpellier, Montpellier, France; g INRAE, UMR CBGP, F-34398 Montpellier, France

**Keywords:** Integrated pest management, Crop pest, Vector-borne disease, Biological control, Agent-based modelling, Population dynamics, Epidemiology

## Abstract

The sterile insect technique (SIT) is an environmentally friendly pest control strategy that consists of inundative releases of mass-reared sterilized males over defined areas, where they mate with wild females, resulting in no offspring and a declining pest population. The technique has effectively managed many crop pests and vector-borne diseases worldwide. A new approach, called boosted SIT, has been proposed to gain efficiency. It combines SIT with the contamination of wild females by sterile males previously coated with biocides. The present study investigated to what extent life history traits of the target pest and biocides can make the boosted SIT more efficient than the classical SIT. We built a generic agent-based model (SIT++) that simulates the population dynamics of insect pests. We then explored parameters related to the mating system, spermatic competition, and fecundity, taking examples from the biology of three well-known Dipteran pest species (*Bactrocera dorsalis, Ceratitis capitata,* and *Glossina palpalis gambiensis*)*.* We found that for boosted SIT to be more beneficial than SIT, horizontal transmission of the biocide to the same generation and to the progeny must be very high. Female fecundity was the other key parameter behind the success of boosted SIT, which was more efficient with insect pests having low reproduction rates. In particular, vertical transmission and late killing time were critical parameters. We also observed that a high level of virulence can help, but only when the boosted SIT is already advantageous; otherwise, it becomes detrimental. The boosted SIT might be advantageous depending on the life history traits of the target pest and transmission routes. For a more extensive exploration, the model can easily be tailored to pests with very different life history traits.

## Highlights

• Sterile males are used as biocide disseminator for insect pest control.
• SIT++ model simulates the combination of mayor pest traits and biocide features.
• Control success requires high biocide horizontal and vertical transmission rate.
• A high lethality of a biocide could emphasize success of the control.

## 1 Introduction

The Sterile Insect Technique (SIT) is a biological control method that is increasingly being used in the framework of insect pest management (Knipling, 1955, 1959). It is environmentally friendly and can successfully reduce crop pests and disease vector insects. SIT is usually a part of an Area-Wide Integrated Pest Management (AW-IPM) program (Hendrichs et al., 2021) that aims to manage pest populations within their functional area to prevent re-infestation due to migration (Klassen and Vreysen, 2021). This involves the mass-rearing and sterilization, using radiation, of a target pest, followed by the release of the sterile males over defined areas, where they mate with wild females, resulting in no offspring and a declining pest population. SIT has been implemented worldwide and successfully controlled many insect pest species from different orders, including Diptera (fruit flies, screwworm fly, tsetse flies, mosquitoes), Lepidoptera (moths), and Coleoptera (Wyss et al. 2000; Ito et al. 2003; Kohama et al., 2003; Orankanok et al., 2007; Boersma, 2021; Gato et al., 2021; Vreysen et al., 2021). As the success of mating between wild and sterile insects is necessary for SIT, it is essential to release large numbers of sterile males to allow this interaction. Thus, implementing SIT in a large area usually requires substantial cost (Mumford, 2021). In this regard, an advanced method called “boosted SIT” could improve the efficiency of SIT and reduce the number of sterile males to release. It was proposed by Bouyer and Lefrançois (2014) and relies on the use of sterile male insects as vectors of a biocide toward conspecifics. The transmission of the biocide occurs mainly during mating (Novelo‐Rincón et al., 2009; Dimbi et al., 2013; Sookar et al., 2014), but also when inoculated males attempt to mate with the wild females without succeeding (Toledo et al., 2014; García-Munguía et al., 2011). For insects such as tephritid fruit flies that exhibit a lekking behaviour, biocide transmission can also happen between sterile inoculated males and wild males in the lek, area where males gather for the sole purpose of attracting and courting females (Emlen and Oring 1977). That phenomenon was suggested by authors like Thaochan and Ngamponsai, (2015, 2018) and Diop et al. (2024) during studies in laboratories and field cages. The use of sterile *Ceratitis capitata* (Wiedemann, 1824) males inoculated with different formulations of *Beauveria bassiana* ((Balsamo-Crivelli) Vuillemin, 1912) has been previously investigated in coffee fields in Guatemala. The sterile inoculated males were able to transmit spores and contaminate at least 44 % of the wild population (Flores et al., 2013; Toledo et al., 2017). However, despite these encouraging tests, there is still a need to clarify many issues for this method, such as the number of males to release or even the conditions for the successful transmission of the biocide. Simulation models are a good way to test the technique as they can generate scenarios that can help reducing the incertitude during pilot field experiments.

Models have been developed to assess the feasibility of the SIT, some of them dealt with mosquitos (Esteva and Mo Yang, 2005; Cai et al., 2014; Multerer et al., 2019), while other models focused on tsetse flies (Barclay and Vreysen, 2011; Dicko et al., 2014), and crop pests like fruit flies and Lepidoptera (Ito et al., 1977; Kean et al., 2011; Potgieter et al. 2013; Barclay et al., 2014). Many models are generic, i.e., they represent a variety of pest and environmental contexts (Barclay, 1982, 2005; Kean et al., 2008). Mathematical modelling has been the most used approach to explore the SIT (Dumont and Tchuenche, 2012; Ben Dhahbi et al., 2020) compared to a few agent-based models (Isidoro et al., 2009; Lin et al., 2015). The questions explored the most aimed at improving the success of SIT by studying the sterile-to-wild male ratios, the mating competitiveness, and the dispersal of sterile males (Ito, 1977; Chargui et al., 2018; Tyson et al., 2007; Dufourd and Dumont, 2013). Regarding the boosted SIT, to our knowledge, only three models have been developed to analyse the conditions of the feasibility of the approach. Pleydell and Bouyer (2019) and Haramboure et al. (2020) proposed mathematical models to explore the use of sterile males as vectors to disseminate the pyriproxyfen to control *Aedes spp* populations. In addition to horizontal transmission during mating, this insecticide is transmitted vertically to larval breeding sites by females, thus it prevents the growth of immature stages. Pleydell and Bouyer and Haramboure et al. found that, compared to the SIT, the boosted SIT reduced mosquito suppression time, required fewer males to be released, and increased the effectiveness of the SIT when sterile males were not very competitive. Diouf et al. (2022) built an agent-based model to compare the efficacy of SIT and boosted SIT to control a population of *Bactrocera dorsalis* (Hendel 1912) fruit flies and reduce fruit infestation in mango orchards. Their results showed that both techniques had the potential to successfully minimize fruit fly abundance and the number of infested fruits, the boosted SIT having a better effect than the SIT alone. These models of boosted SIT are specific to a pest-biocide combination and eventually are intended to be used for a defined landscape context. Their application to other pest species, biocide type, or landscape context would require essential modifications. A more generalized approach should consider the main features of the system, including the target pest and the biocide. It could enable to bypass the lack of knowledge when it comes to the interactions between specific pest and biocide, and also be applied to various pest and landscape types. Such a generalized model could give guidelines for choosing the suitable type of biocide and general rules for the success of a boosted SIT program for any given pest system.

In the present study, we propose a generic model to explore the conditions of success of the boosted SIT in controlling a diversity of pest insects. For this purpose, we built an agent-based model (ABM), taking into account an essential aspect of the success of the boosted SIT: the interactions between individuals of a pest population and a biocide type. ABMs explicitly describe a system's unique and autonomous entities as discrete and specific agents. Agents can decide and adapt their behaviour according to their state and environment. ABMs can be applied to several areas of biology, including those concerned with pest invasions (Railsback and Grimm, 2019). In this paper, we present a generic ABM to represent pest flies from different species, including fruit flies (Diptera, Tephritidae) and tsetse flies (Glossina). These insects vary in terms of significant life history traits such as mating systems, spermatic competition, and fecundity. We simulated the impact of SIT and boosted SIT scenarios on fly abundance according to their life cycle and the biocide type, including transmission and induced mortality.

## 2 Material and methods

### 2.1 Model description

The simulation model was named SIT++. Its description follows the Overview, Design concepts, Details (ODD) protocol (Grimm et al., 2006, 2010, 2020) for describing individual- and agent-based models. The model was developed using the Netlogo 6.2 platform (https://ccl.northwestern.edu/netlogo/6.2.2/).

#### 2.1.1 Purpose

The objective of SIT++ is to identify the conditions for the success of the boosted Sterile Insect Technique in controlling a population of a given insect pest. Here, insect pests include disease vectors and crop pests. More specifically, the model simulates the effect of the boosted SIT on the population dynamics of a given insect pest, considering the biocide type and life history traits.

#### 2.1.2 Entities, state variables and scales

The model represents a single type of entity, i.e., the individuals of the same insect species for a given simulation. Individuals are characterized by states variables (cf. Table 1) as their sex (*male/female*), developmental stage (*immature/mature*), age, time in immature stage (*Timmature*), generation to which they belong, health status (*healthy, contaminated or dead*). Each female has a probability of mating and reproducing depending on her sexual maturity and the number of mating to execute (*Nmating*). She also has a list that keeps the history of the mating partners according to their reproductive status (sterile = 0 or fertile = 1) and the total number of male and female eggs to be produced following mating. A male, on the other hand, has a competitiveness value for mating (*Csterile* for sterile males and *Cwild* for wild males) as well as a reproductive status of *sterile* or *fertile.*

**Table 1:** List of parameters of the SIT++ model.

Name	Value	Description
*Population*		
*N_0_*	30	The initial number of individuals
*Tgeneration*	60	Lifetime of individuals (days) (could theoretically be >100)
*Pimmature*	33	Lifetime in immature stages (%)
*Females*		
*F*	6 - 45	Number of immature individuals produced per female
*N_mating_*	3	Number of matings performed by females throughout their lifetime
*Males*		
*Csperm*	first – last - share	Spermatic competition
*Csterile*	0.5 – 1 – 2	Mating competitiveness of sterile males
*Cwild*	1	Mating competitiveness of wild males
*Ratio*	1 – 20	The ratio of the number of sterile males released to the number of wild males
*Biocide*		
pH	0.1 – 0.25 – 0.5	Probability of horizontal transmission of the biocide during mating
*P_v_*	0 – 0.25 – 0.5	Probability of vertical transmission of the biocide
*LT*	0.1 – 0.25 – 0.5 – 0.75 – 0.9	Lethal time of the biocide (proportion of the time in the mature phase)
*Virulence*	0.25 – 0.5 – 0.75 – 1	Probability of the biocide to kill the contaminated individual at the end of *LT*
*P_c_*	0 – 0.25	Probability of horizontal transmission of the biocide during lekking (contact between males and mating attempts)

The time step of the model is one day. The time horizon of the simulations is several days depending on the generation time of the insects and the number of generations needed to reach a problematic population size, noted as *N_X_* (conceptually considered to be the size when the insect population is an issue for human, animal or plant health if no control measure is applied, here 1000 times the initial population size *N_0_* fixed at 30, Table 1). Two crucial time steps have been defined in the simulated population dynamics: step *t* and step *t* *+* *1*. Step *t* *+* *1* is defined as the time when the population size reaches *N_X_* without control while *t* is the time when adults of the generation preceding the one whose population size was problematic, start to appear in the population. The time *t* is also the time the release of sterile males is performed to control the population dynamics, as we considered that these adults were the individuals that could lead the population to *N_X_*.

#### 2.1.3 Process overview and scheduling

Each time step of the model simulations was subdivided into three main parts: (1) agents grow up, (2) they reproduce, and (3) they can change their health status. These three parts varied depending on the simulation scenario: (i) no control measure, (ii) release of sterile males (SIT), and (iii) release of sterile males carrying a biocide (boosted SIT). Thus, at each time step, the model simulation considered the following eight processes:

1. Each agent goes through stages of development before becoming an adult (age > *Timmature*, see Table 1).
2. Under the boosted SIT scenario, contaminated agents update their time since contamination *Tsick* by 1. When *Tsick* ≥ *Tcontamination*, a *Virulence* probability (Table 1) of dying and 1-*Virulence* of healing are calculated.
3. Under the SIT or boosted SIT scenarios, the release of sterile males occurs when the time counter is at *t*. Then, the number of sterile males to be released is computed as the number of wild adult males in the population multiplied by a *ratio* set between 0 and 20 (Table 1). This procedure is performed only once in the simulation, just before reproduction, but after development to consider all individuals in the population that can participate in the reproduction.
4. Under the boosted SIT scenario with insects performing leks, contaminated individuals contaminate a certain proportion of the population before reproduction, according to a probability of transmission *P_c_* (Table 1).
5. When mature males and females are available for mating, (1) they search for a mature partner of the opposite sex (see SM1). Subsequently, (2) they carry out a stage of fertilization, and finally, (3) the new generation of immature individuals is produced with an *F/Nmating* number of individuals generated by females (Table 1). When females mate with a sterile male, they do not produce offspring. Their mating history (with wild or sterile males) and spermatic competition will also affect their offspring size (see SM 2).
6. Under the boosted SIT scenario, contaminated females of some insects can contaminate the immature individuals across the laying sites. In this case, it is based on a probability of vertical transmission (*P_v_*, Table 1).
7. Under the boosted SIT scenario, individuals also update their health after mating. Healthy individuals mating with a contaminated one also become contaminated with a probability of transmission pH (Table 1, e.g., females mated with contaminated sterile males).
8. Individuals die after completing their entire lifespan (*age >Tgeneration).*

#### 2.1.4 Design concepts

##### 2.1.4.1 Basic principles

The SIT++ model simulates major processes involved in the population dynamics of insects, but competition for resources (e.g., food and oviposition sites) in the environment was not considered. For simplification, natural mortality of immature stages was also included in the fertility. In other words, the offspring number (fertility *F*) represents the number of immature individuals that reach sexual maturity. Regarding the progression of the disease induced by the biocide in the population, contaminated individuals who survive to the end of the lethal time are considered cured. They are back to a ‘healthy’ state but have not acquired immunity to the biocide and might become contaminated again with the same probability.

##### 2.1.4.2 Interactions

At the time of reproduction, females choose their partner according to their relative mating competitiveness (see SM 1). Contaminated individuals can contaminate others during the lekking and mating phases.

##### 2.1.4.3 Stochasticity

SIT++ included randomness at the initialization level (cf. initialization section) and in the agent processing order (random by default). There are also random draws in biocide transmission procedures between individuals (including transmission by contact between males during a lekking, mating phase, and transmission from females to their offspring) and the male/female ratio of a new generation.

##### 2.1.4.4 Observations

At the end of each time step, the total population size, the proportion of population size to *N_X_*, the number of sterile males, the distribution of individuals by generation, and the success of the control measure (cf. simulation plan below) are recorded. Population size proportions to *N_X_* are calculated at *t* and *t* *+* *1* as follows:

$$pN_{t+1}=\frac{N_{t+1}}{N_{0}\times 1000}=\frac{N_{t+1}}{N_{X}}$$

where $N_{t+1}$ is the size of the population at time *t* + 1 and $N_{0}$ the initial population size (Table 1).

#### 2.1.5 Details

##### 2.1.5.1 Initialization

At initialization of each simulation, the time spent in the immature stage (*Timmature* Table 1) is calculated from the time needed for a complete generation (*Tgeneration*, Table 1) and the proportion of lifetime in the immature stage (*Pimmature*, Table 1). The health status of insects (*N_0_* with an equal number of males and females, Table 1) is initialized to ‘healthy’ with generation 0. Different ages are assigned to individuals following a uniform distribution to represent overlapping generations. Individuals do not have a pre-assigned mating partner. The number of mating is set for females, while males have no number or time limit for mating. Males are fertile and have an assigned spermatic competition, and their mating competitiveness is set to 1 (*C* = 1, Table 1). Females are available for mating and have not performed any mating at the start of the simulation. Their fecundity *F* and their maximum number of matings are fixed (Table 1). The number of expected generations is determined by:

$$nbGeneration=\frac{-\ln1000}{\ln2-\ln F}$$

where *F* is the number of immature individuals produced by one female during her lifetime, ‘1000′ being the multiplication factor from *N_0_* to *N_X_* and ‘2′ the balanced sex-ratio. This number of expected generations to reach *N_X_* without control measures allowed us to anticipate when *t* + 1 should occur and, hence, determine when would be *t*, the timing of the release of sterile males in SIT and boosted SIT scenarios.

The list of females of breeding age was determined by dividing the duration of the adult stage by the number of matings that the female must perform.

##### 2.1.5.2 Inputs

No forcing data were used during simulations.

#### 2.1.6 Submodels

##### 2.1.6.1 Partner choice and male competitiveness SM1

All males had a competitiveness for mating. Wild males had a competitiveness of 1, whereas that of sterile males varied from 0.5 to 2 (*Csterile*, Table 1). The competitiveness of sterile male is usually lower than that of wild male but we assumed that for some insect it can be higher when the sterile males received a diet before being released (Orankanok et al., 2013; Adnan et al. 2020).

Males participating in reproduction were more likely to be selected by females when their competitiveness was high. Males were drawn by females from a list of probabilities constructed by attributing to males the ratio of their competitiveness to the sum of the competitiveness of the male population. A cumulative probability vector was constructed by adding this competitiveness ratio for each male to that of the previous males in the list. Thus, a male was chosen if the number of the draw was within the probability interval between the last male in the list and him. The stronger his competitiveness, the greater the interval and the chance of being chosen.

##### 2.1.6.2 Spermatic competition SM2

The spermatic competition was simulated by the calculation of the number of eggs, for each laying event, according to the history of the mating of the female. The model considered three cases of spermatic competition: (1) «First» which considered that the first partner of a female would be the contributing male for all the offspring; (2) «Last» which was the opposite, meaning the last partner of a female would be the contributing male for all the offspring to be produced; (3) «Share» which represented an equitable sharing between the different partners preceding a laying event of a female.

Females kept their matings' history, corresponding to a list of 0 (= sterile) and 1 (= fertile). This allowed to keep a counter of the number of fertile male partners useful for the calculation of the number of eggs in the case of « Share ». Depending on the case of spermatic competition, the calculation of the number of eggs produced for a laying event was computed as follows:

$$Neggs\;=\left\{ \begin{array}{c} \frac{F}{N_{mating}},\;for\;cases\;\text{first}\;and\;\text{last}\\ moy\times \frac{F}{N_{mating}},\;for\;cases\;\text{share} \end{array}\right.$$

Where *F* is the fertility of females (Table 1), *Nmating* the number of matings females perform during their lifetime. For the case “first”, if the 1st male partner was sterile (= 0), females produced only unfertilized eggs. For the case “last”, if the last partner was sterile, the female produced unfertilized eggs for the ongoing reproduction event. For the case “share”, the distribution of the number of eggs to be produced was determined by the number of fertile males with whom the female had mated divided by the total number of matings she had performed (moy computed as the average of 0 and 1 from the list of mating partners history). In all competition cases, the number of eggs produced was then evenly distributed between males and females for the generation of the offspring.

##### 2.1.6.3 Probability of horizontal and vertical transmission SM3

During mating, when healthy individuals that had contact with a contaminated individual also became contaminated according to a probability of horizontal transmission *P_h_* (e.g., females that mated with a contaminated male, healthy males that mated with a contaminated female). If the vertical transmission is considered, the contaminated females produce offspring that are contaminated according to a probability of vertical transmission *P_v_*. In the case where males performed leks (*P_c_* > 0), following a probability of transmission by contact *P_c_*, a contaminated individual contaminated another individual with probability *P_c_*.

### 2.2 Simulation plan

We explored different parameters of the model to evaluate under which conditions the boosted SIT could be more successful than SIT, considering generic life history traits inspired by the fruit flies *B. dorsalis* and *C. capitata,* and the tsetse flies, *Glossina palpalis gambiensis* (Vanderplank 1911). The parameters explored are the fecundity of females, the mating competitiveness of sterile males and the spermatic competition as they can be very different between fly species. Four explored parameters concerned the biocide for boosted SIT: virulence, lethal time, and horizontal and vertical transmission probability. One parameter concerned the competitiveness of sterile males, which could vary depending on breeding conditions and release methods. Two parameters also explored, concerned the target insect pest with low or high fecundity of females and lekking behaviour that would allow horizontal transmission of the biocide among males (Table 1).

Three scenarios were simulated for each combination of parameters: no control treatment (C), SIT and boosted SIT. These scenarios were launched with the same initialization conditions thanks to an initialization seed of the generated random number chain that kept the same values of stochasticity between the scenarios.

The first scenario (C) stopped when the population size reached the problematic ***N_X_***. The number of days to reach this problematic population size (***t*** ***+*** ***1***) was stored for use in the two other scenarios. Subsequently, the SIT and boosted SIT scenarios were repeated with different values of sterile/wild male ratio, starting with a ratio of 1 and increasing by one unit to 20. After the stored number of days (*t* + 1), the population size was then recorded to assess the effect of the SIT and boosted SIT (*N_t+1__SIT* & *N_t+1__SIT_b*). These scenarios stopped when the population size at *t* *+* *1* (*N_t+1__SIT* or *N_t+1__SIT_b*) was inferior to ***0.5 N_X_*** (see supplementary material S1) or if the maximum value of 20 for the sterile/wild male ratio was reached without success. As soon as these conditions were reached, the sterile/wild male ratio that allowed the success was recorded (*ratioSIT* or *ratio_SIT_b* for the SIT and boosted SIT scenarios, respectively). Then, the ratio (***G***) was calculated with the sterile/wild male ratios that enabled SIT and boosted SIT to be successful:

$$\text{with}G=\frac{ratio\_SIT\;-\;ratio\_SIT\_b}{ratio\_SIT\;+\;ratio\_SIT\_b}$$

For each simulation, 50 replications were performed to consider the stochasticity present at initialization. The final ratios (*ratio_SIT, ratio_SIT_b, and G*) for each parameter combination were averaged over the 50 replicates.

Two situations were considered to analyse the results. (1) The SIT and boosted SIT succeeded, but the boosted SIT allowed the reduction of the number of sterile males to release (*G* > 0). In other words, although SIT did not fail, the effect of an epidemic with the boosted SIT made it possible to succeed while decreasing the number of sterile males needed to be released. (2) The boosted SIT succeeded while the SIT failed. In this case, we looked at the ratio of the number of replicates where the boosted SIT satisfied the condition of success (*N_t+1__SIT_b* < 0.5 *N_X_*) over the number of replicates when the SIT did not (*N_t+1__SIT* > 0.5 *N_X_*). To visualize the results with the G value for these situations where SIT failed, the G was set to 1, representing the advantageous contribution of the boosted SIT.

## 3 Results

**Fig. 1:**
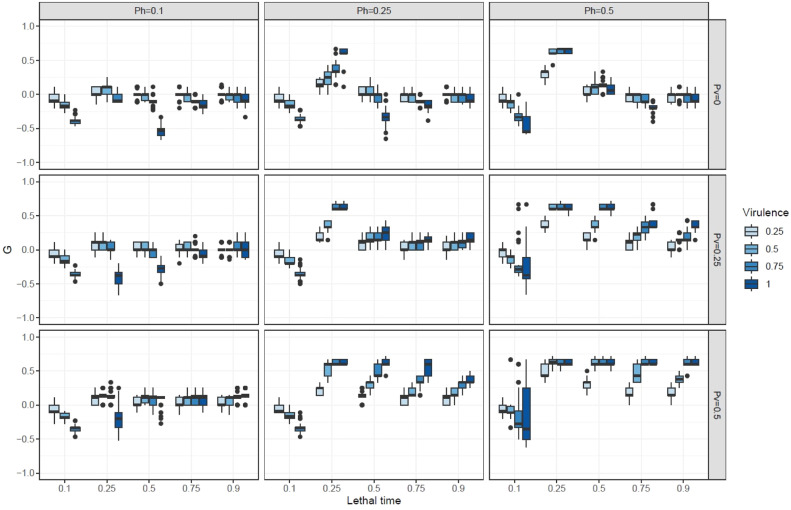
Ratio of boosted SIT success compared to SIT (*G*) in relation to the biocide's lethal time, virulence (blue levels), the probability of transmission during mating and to offspring (*Ph and Pv*) for fixed fecundity of females (*F=6*), mating competitiveness of sterile males (*Csterile = 0.5*), spermatic competition (*Csperm = “first”*), and transmission of biocides in leks (*Pc = 0.25*).

For fixed fecundity of females (*F* = 6), mating competitiveness of sterile males (*Csterile* = 0.5), spermatic competition (*Csperm* = “first”), and transmission of biocides in leks (*Pc* = 0.25), the higher the transmission of the biocide (horizontal or vertical), the higher the chances to observe the success of boosted SIT over SIT (Fig. 1). More specifically, we observed that with no vertical transmission (top row of Fig. 1), the transmission during mating needed at least pH = 0.25 to obtain a gain. In that case, the lethal time should not be too high (*LT* = 0.25), and the higher the virulence, the greater the gain. The restricted intermediate lethal time results from a trade-off between (1) enough time to transmit the biocide to the highest number of wild males before death and (2) the need to kill mature individuals before reproduction. In other cases, the boosted SIT did not bring significant benefits. When vertical transmission occurred (*Pv* > 0), the range of lethal time increased to 0.75, meaning that the longer time for the biocide to have a lethal effect was balanced by contamination of the next generation.

**Fig. 2:**
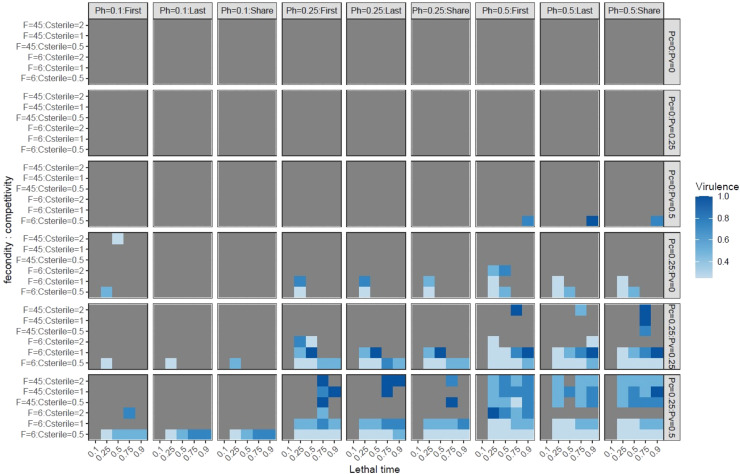
Minimum virulence values (blue levels) for the success of boosted SIT against SIT (*G>0.1*) depending on the female fecundity (*F*), the competitivity of sterile males (*Csterile*), the lethal time, the spermatic competition (*first, share, last*), the transmission rate during mating and to offspring (*Ph, Pv*), and the transmission rate in lek (*Pc*).

Exploring all fixed parameter values led to 35 other figures as Fig. 1 (see supplementary material S2). These simulation results are summarized in Fig. 2 in the form of the minimum virulence of the biocide needed to have a significant benefit of the boosted SIT over SIT in terms of sterile/wild male ratios (*G* > 0.1). In Fig. 1, when *G* < 0, virulence had a negative effect on *G*. On the contrary, when *G* was above 0, virulence positively affected *G*. Hence, looking at the minimum virulence values for the success of boosted SIT in Fig. 2 does not hide the benefits of the boosted SIT over the SIT.

Without transmission of the biocide among males during leks (*Pc* = 0), the boosted SIT was rarely more successful than SIT (Fig. 2). Only in extreme situations of high horizontal and vertical probability of transmission (pH = *Pv* = 0.5), high virulence of the biocide (darkest blue), long lethal time (*LT* = 0.9), low fecundity of females (*F* = 6) and low mating competitiveness of sterile males (*Csterile* = 0.5), the boosted SIT was more successful than SIT. These results supported the idea that biocide transmission within the target pest population is critical to the success of the boosted SIT. Female fecundity also played a significant role in the success of the boosted SIT. With no vertical transmission and enough horizontal transmission (*Pv = 0, Pc* = 0.25, pH ≥ 0.25), high fecundity (*F* = 45) never led to a gain of success of boosted SIT. In such a situation, only low fecundity (*F* = 6), in some situations of intermediate lethal time and relatively low mating competitiveness of sterile males, was advantageous for the boosted SIT. When vertical transmission increased (*Pv* ≥ 0.25 still with *Pc* = 0.25, pH ≥ 0.25), then high fecundity (*F* = 45) situations gave better control of boosted SIT over SIT. Here again, these results showed that for boosted SIT to be an advantage compared to SIT, the biocide needs to be very well spread within the population. The effect of fecundity, in the absence of vertical transmission, illustrates the assumption that the faster the population dynamics of an insect, the more transmissible the biocide needs to be for the boosted SIT to be efficient.

Overall, we did not observe the effects of spermatic competition regimes on the success of the boosted SIT. The low mating competitiveness of sterile males was generally more likely to have situations of positively boosting SIT, as the biocide-induced mortality probably balanced the low rate of sterility induction with SIT.

We found a few cases (only 8 %) of unsuccessful SIT corresponding to high fecundity (*F* = 45) and low mating competitiveness of sterile males (*Csterile* = 0.5). In these cases, the boosted SIT could be successful depending on the biocide's lethal time and the probability of horizontal transmission (Fig. 2). In such cases, we verified our simulation results and had 100 % of simulations with boosted SIT, successfully reducing the target pest population.

## 4 Discussion

The SIT++ simulation model has been developed to evaluate the efficacy of control measures against insect pest populations under different scenarios of SIT and boosted SIT. Another objective was to assess under which conditions, particularly parameters of the target pest life history traits, biocide virulence and lethal time, and transmission of the biocide, the boosted SIT allows better control than SIT.

We demonstrated the sensitivity of its outcomes to some critical parameters of life history and insect-pathogen interactions. For the boosted SIT to be more advantageous than the SIT, horizontal transmission of the biocide was critical, not only through female-male interactions during mating, but also at mating attempts and contact between males during lekking. Boosted SIT had to be placed in conditions of very high pathogenicity to be efficient, but only when boosted SIT is already advantageous; otherwise, it becomes detrimental. Female fecundity was the other key parameter behind the success of boosted SIT, which was more efficient with insect pests having low reproduction rates.

The combination of high horizontal and vertical transmission allowed the success of the boosted SIT over SIT. These results confirm those found with the boosted SIT using pyriproxyfen to control mosquitoes (Haramboure et al. 2020). We showed that the boosted SIT has a better chance of exceeding the SIT when vertical transmission occurs. In the case of entomopathogenic fungi (*M. anisopliae* or *B. bassiana*) as biocides to control tephritid fruit flies, for example, vertical transmission to eggs and early larval stages has not been observed. However, microbiomes are transferred vertically in Drosophila flies (Guilhot et al., 2023). Therefore, further research on the vertical transmission of pathogens on tephritid fruit flies is needed. Additionally, these fungi can reduce female fecundity, as demonstrated in the fruit flies *C. capitata, C. cosyra*, and *C. fasciventris* (Quesada-Moraga et al. 2006; Dimbi et al. 2013). Concerning the pupariating larvae and pupae, Ekesi et al. (2002) and Ekesi et al. (2011) showed that their exposure to contaminated soil reduced the number of emerging adults. Thus, soil inoculation with entomopathogenic fungi under the host plants, in addition to the horizontal transmission, could support the efficacy of the boosted SIT compared to the SIT.

Our model showed that transmission of the entomopathogen between males during lek and during the mating attempts are very important for the success of the boosted SIT. This result was consistent with the field study conducted by Toledo et al. (2017). They used sterile males of the Mediterranean fruit fly, *C. capitata*, as disseminators of *B. bassiana* to the wild population in coffee-growing areas and autoinoculation devices in Guatemala. They observed that the release of sterile males was more effective in terms of spores’ transmission, suggesting a high transmission rate during male aggregations (leks). Despite authors’ deduction about this male-to-male transmission (Toledo et al. 2017; Diop et al., 2024), there are no studies that has measured it as far as we know. Further attempts to implement the boosted SIT in the field should focus on insect species that display a form of lek or aggregation behaviour to allow the possibility for contaminated males to transmit their biocide to male congeners before mating. These behaviours are sometimes unknown in natural conditions, and further research on male-male interactions in the field is often needed to discover behaviours that were never noted in laboratory settings (Maeno et al., 2021).

We observed that an increase in virulence of the pathogen may benefit the boosted SIT when it is more efficient than SIT. This is an unexpected result as previous authors associated a high virulence with a lower efficiency of insect control using inoculated sterile males (Toledo et al., 2017). This was seen in our simulations when SIT was more efficient than boosted SIT: the increase in lethality decreased the effect of the boosted SIT. The corollary of these findings could be that if, during experiments of the boosted SIT, an increase of pathogenicity does not reduce the pest population size, this means that the boosted SIT is probably less suitable than the classical SIT.

Temporal fluctuations of population size ahead of the application of SIT or boosted SIT have not been considered in our model. Most insect pests show seasonal variations in population size which may be linked to the availability of resources or climatic conditions (Diatta et al. 2016; Thiemann et al. 2011; Martay et al. 2016). Considering these environmental conditions for specific insect species would allow to optimize SIT or boosted SIT by finding the best period to make sterile male releases. It is well-known that SIT practitioners always ensure populations are at low density when using the technique (Knipling, 1959; Vreysen, 2001). In our model, releases were made when the fly population was growing but had not reached an arbitrary threshold of tolerable economic losses. A single release of sterile males was carried out in each simulation, and this led to an immediate reduction in the population. In other words, we estimated the instantaneous effect of SIT and boosted SIT on population growth rate and postulated that this effect was independent of the demographic context. The model of Diouf et al. (2022) follows a different rationale: they simulated repeated releases that could be initiated in various demographic contexts, enabling them to propose different periods for releasing sterile males. Another more recent study by our group explored the frequency and number of releases (Diouf et al. in press) and found that earlier and more frequent releases lead to better SIT or boosted SIT controls. These two models were more case-specific than the present study, hence permitting the consideration of demographic seasonality and insect release temporality. These temporality questions need to be explored for specific insect systems.

We must point out a caveat to our study in that mortality caused by the biocide was independent of the transmission route (e.g., donors and recipients) and the type of insect considered (e.g. sterile and fertile). Depending on the case of pest and biocide, there is sometimes higher mortality in donors who generally receive greater doses than in recipients, and we can have faster mortality in males than in females, and vice versa (Dimbi et al., 2013; Sookar et al., 2014). Models such as that of Haramboure et al. (2020) and Diouf et al. (2022) incorporated these differences in mortality rates. However, we have chosen to generalize this aspect in our model, given that we are simulating several pest species and biocides.

To our knowledge, the models that have explored the success of the boosted SIT were interested in biocides whose pathogenic and transmission properties were already studied in laboratory or field conditions in the framework of pest control. We have the example of pyriproxyfen considered in the model of Pleydell and Bouyer (2019) and Haramboure et al. (2020) for the control of *Aedes albopictus* (Chandel et al., 2016; Unlu et al., 2017) or the *M. anisopliae* considered in Diouf et al. (2022) for the control of *B. dorsalis* (Tora and Azerefegn, 2021). Unlike these models, the SIT++ model provides information based on pest and biocide traits to promote the success of the boosted SIT. This is a significant result that can guide laboratory research on selecting the right biocide features: efficient transmission must occur at low doses and lead to host death. In contrast, high doses should not reduce the host lifespan (i.e., keep a long lethal time) to allow sufficient transmission from mass-reared insects to wild conspecifics.

The boosted SIT did not appear as a universal improvement relatively to classical SIT but may be advantageous in specific contexts. Our simulations showed that the boosted SIT has potential when a suitable biocide, with the right life-history features (e.g., male-male transmission), is used against host populations with compatible phenotypes and behaviours (e.g., leks). More detailed studies, considering, for example, the environment and the demographic context in which the biocide and the target pest interact, would enable us to better guide control measures. For this paper, our model simulated behavioural and developmental traits inspired by fruit and tsetse flies. It could, however, be easily tailored to many other insect pests because it is based on simple rules and parameters representing life-history traits and behaviours.

## Funding

This work has been carried out with the financial support of a Long-term EU- Africa research and innovation Partnership on food and nutrition security and sustainable Agriculture (LEAP-Agri), project Pest-Free Fruit, in the framework of the European Union's Horizon 2020 research and innovation programme under grant agreement No 727,715.

## Credit authorship contribution statement

**Fanny Herbillon:** Conceptualization, Methodology, Software, Formal analysis, Writing – original draft, Visualization. **Esther Gnilane Diouf:** Conceptualization, Writing – original draft, Supervision. **Thierry Brévault:** Conceptualization, Writing – review & editing, Project administration, Funding acquisition. **Marion Haramboure:** Conceptualization**. Simon Fellous:** Writing – review & editing. **Cyril Piou:** Conceptualization, Methodology, Software, Formal analysis, Writing – original draft, Supervision, Visualization.

## Declaration of competing interest

The authors declare that they have no known competing financial interests or personal relationships that could have appeared to influence the work reported in this paper.

## Data Availability

Our model source code is freely available on Cirad Dataverse at https://doi.org/10.18167/DVN1/VFK8UF

## Appendix Supplementary materials

Image, application 1

Image, application 2

Image, application 3
