# Supplementary material for: Life history traits of the target pest and transmission routes of the biocide are critical for the success of the boosted Sterile Insect Technique
Source: Curr Res Insect Sci. 2024 Nov 12;6:100101. doi: 10.1016/j.cris.2024.100101 (PMC11612786; doi:10.1016/j.cris.2024.100101)
Supplement: Supplementary file 1 [file mmc1.pdf]

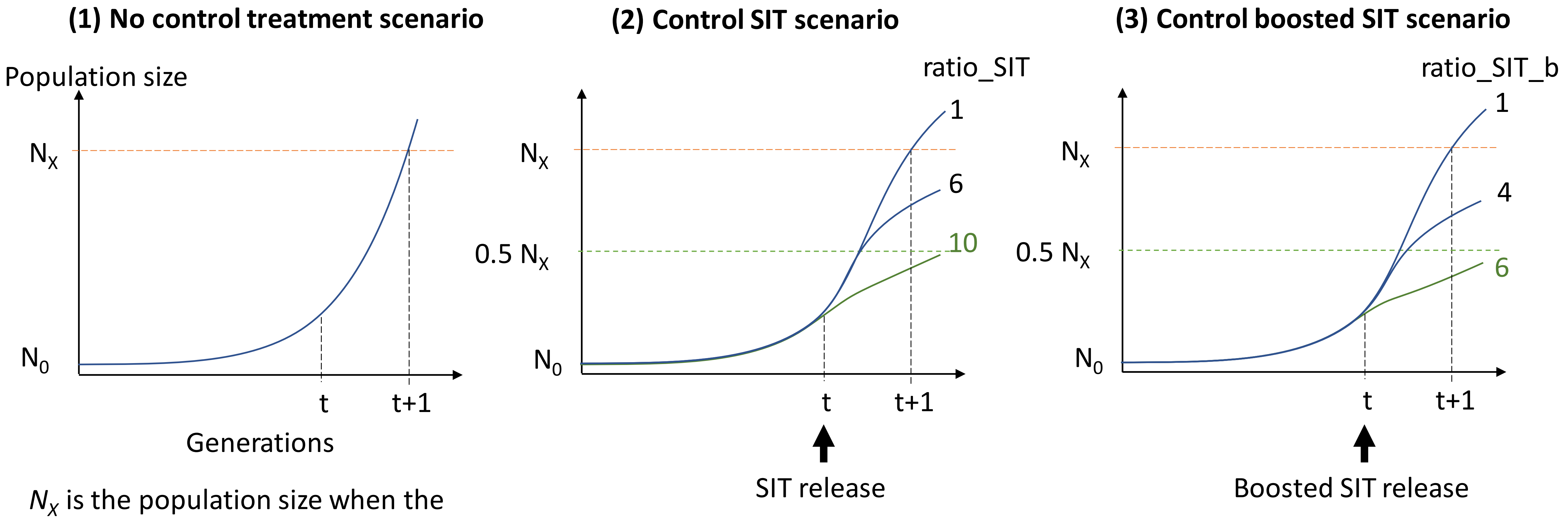

N_X is the population size when the insect population is an issue for human, animal or plant health if no control measure is applied.
Here, we fixed N_0 =30 and
N_X = 1000 x N_0 = 30 000

Success if $N_{t+1} < 0.5\ N_X$
In the above example,
ratio_SIT = 10

Success if $N_{t+1} < 0.5\ N_X$
In the above example,
ratio_SIT_b = 6
G = (10-6)/(10+6)=0.25
