# Supplementary material for: Life history traits of the target pest and transmission routes of the biocide are critical for the success of the boosted Sterile Insect Technique
Source: Curr Res Insect Sci. 2024 Nov 12;6:100101. doi: 10.1016/j.cris.2024.100101 (PMC11612786; doi:10.1016/j.cris.2024.100101)
Supplement: Supplementary file 2 [file mmc2.pdf]

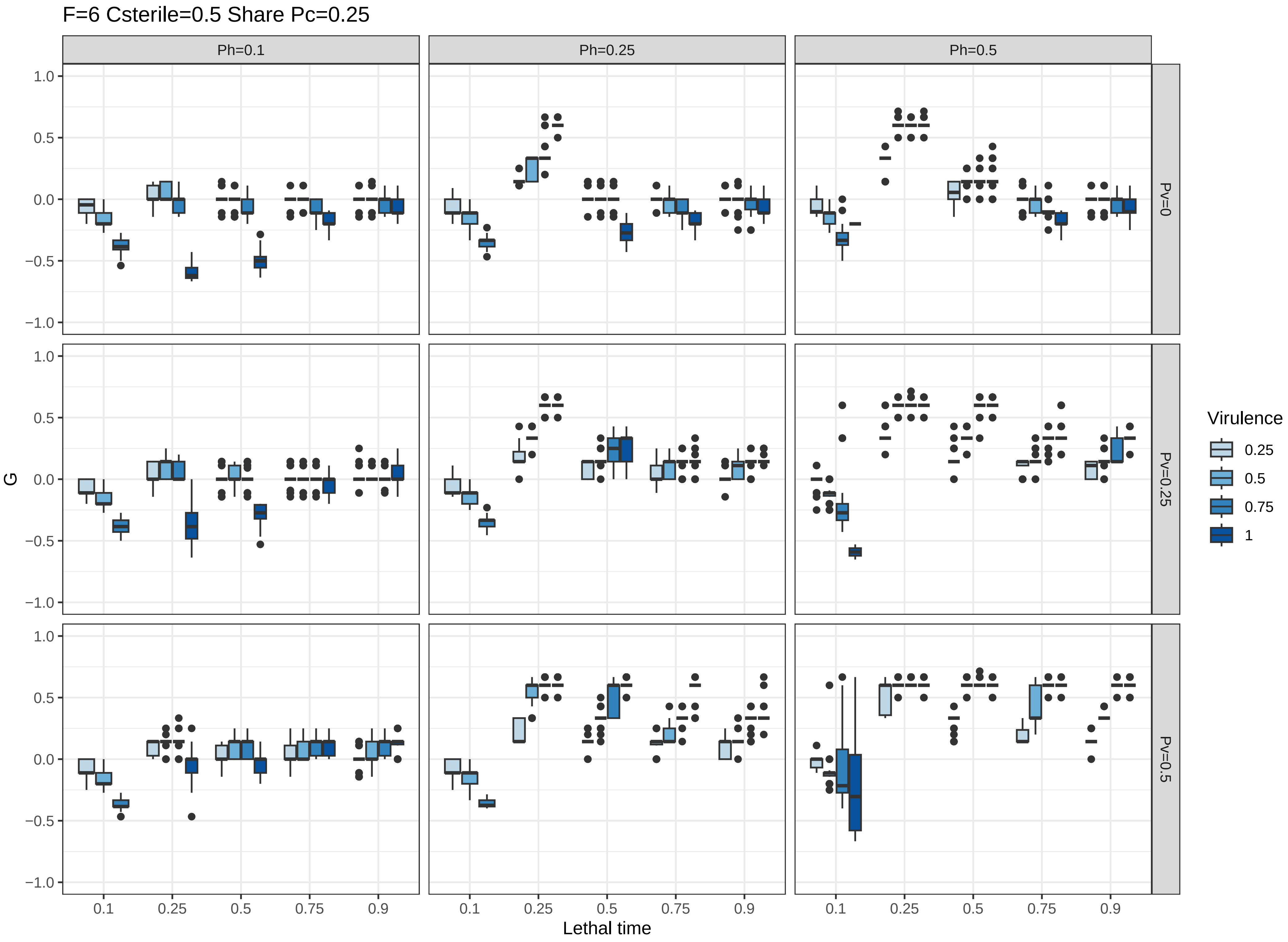

F=6 Csterile=0.5 Share Pc=0.25
Ph=0.1
Ph=0.25
Ph=0.5
Pv=0
Pv=0.25
Pv=0.5
G
1.0
0.5
0.0
−0.5
−1.0
0.1
0.25
0.5
0.75
0.9
Lethal time
Virulence
0.25
0.5
0.75
1

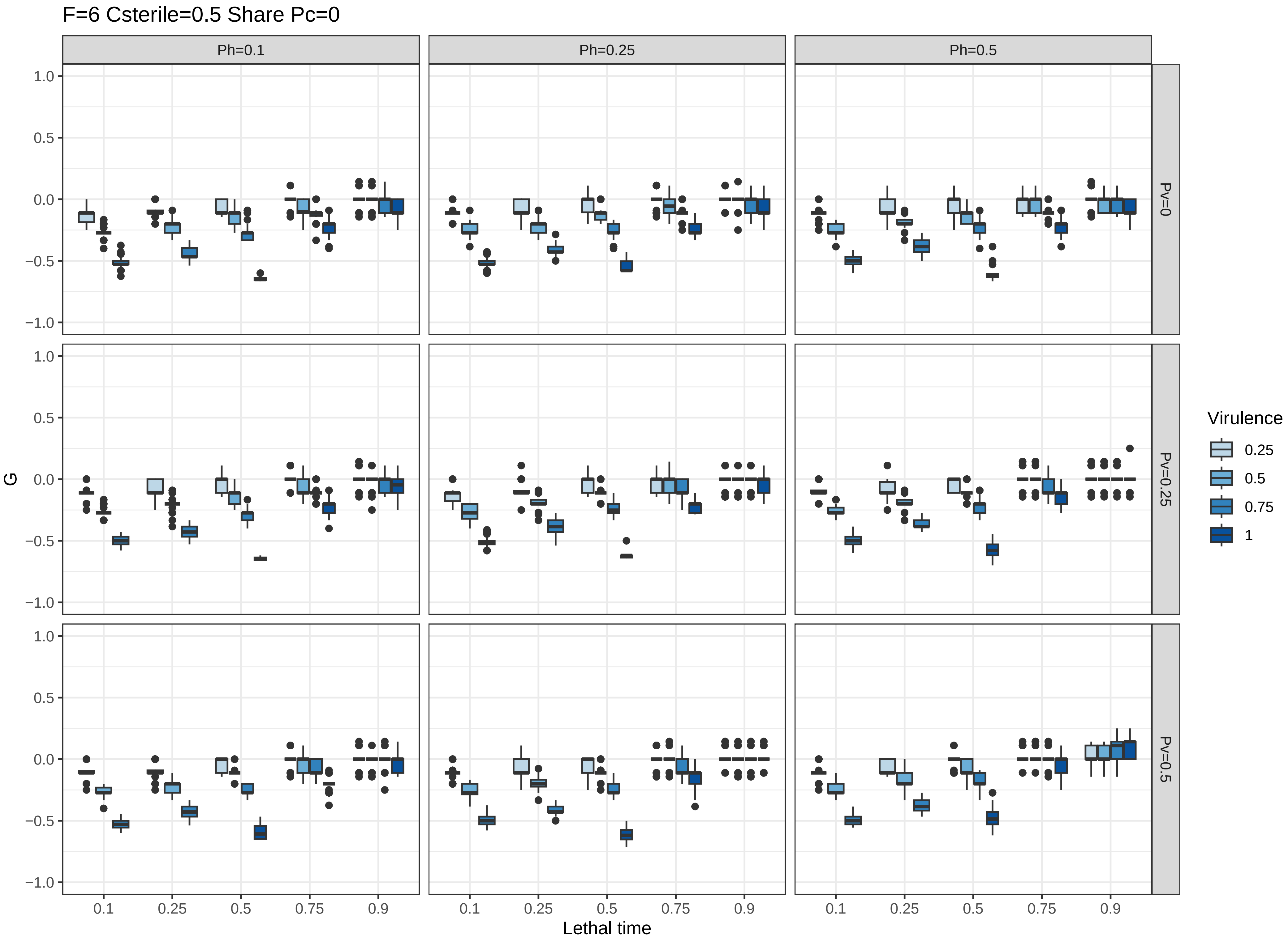

F=6 Csterile=0.5 Share Pc=0
Ph=0.1
Ph=0.25
Ph=0.5
Pv=0
Pv=0.25
Pv=0.5
G
1.0
0.5
0.0
−0.5
−1.0
0.1
0.25
0.5
0.75
0.9
Lethal time
Virulence
0.25
0.5
0.75
1

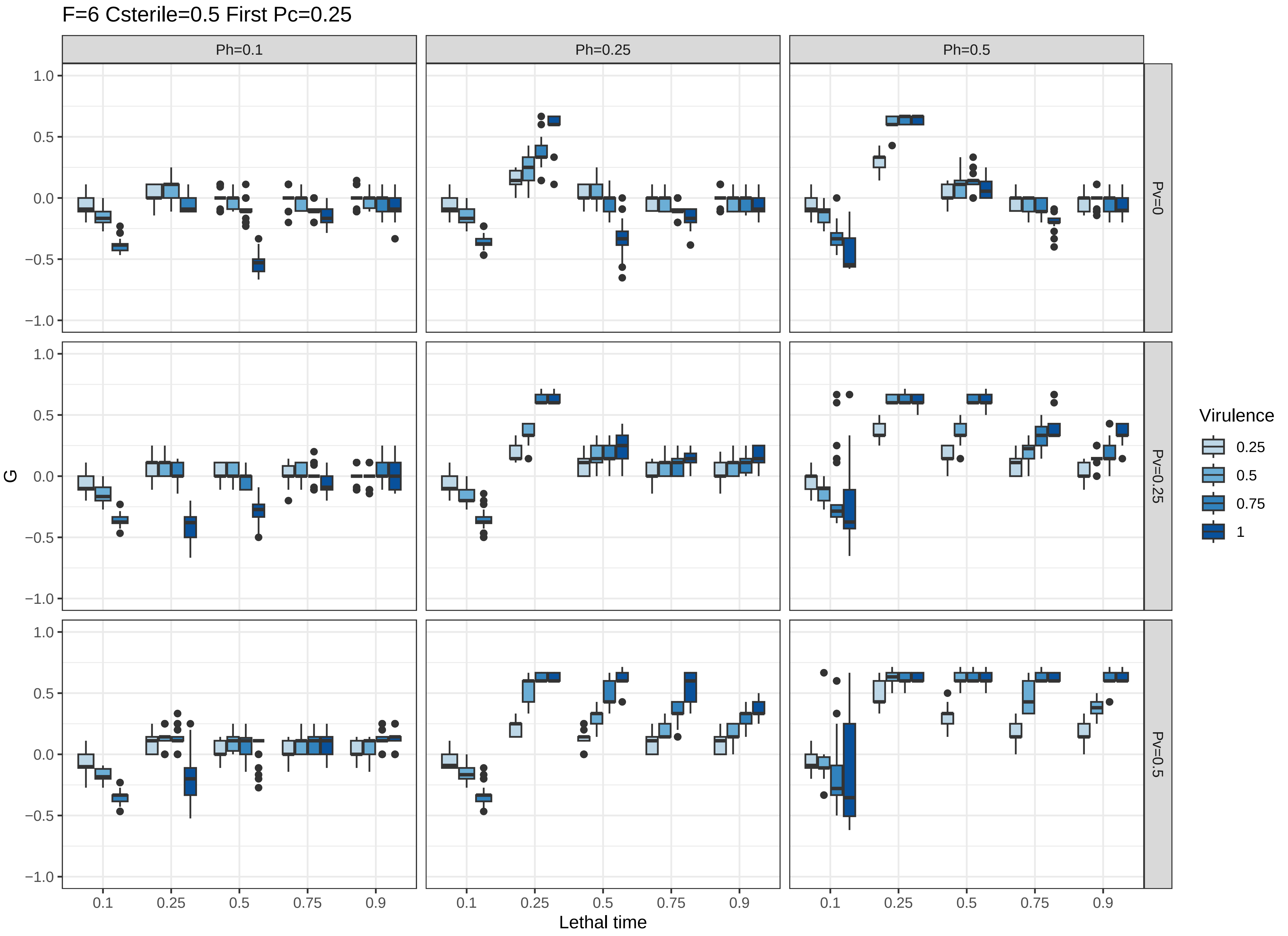

F=6 Csterile=0.5 First Pc=0.25
Ph=0.1
Ph=0.25
Ph=0.5
Pv=0
Pv=0.25
Pv=0.5
1.0
0.5
0.0
−0.5
−1.0
G
0.1
0.25
0.5
0.75
0.9
Lethal time
Virulence
0.25
0.5
0.75
1

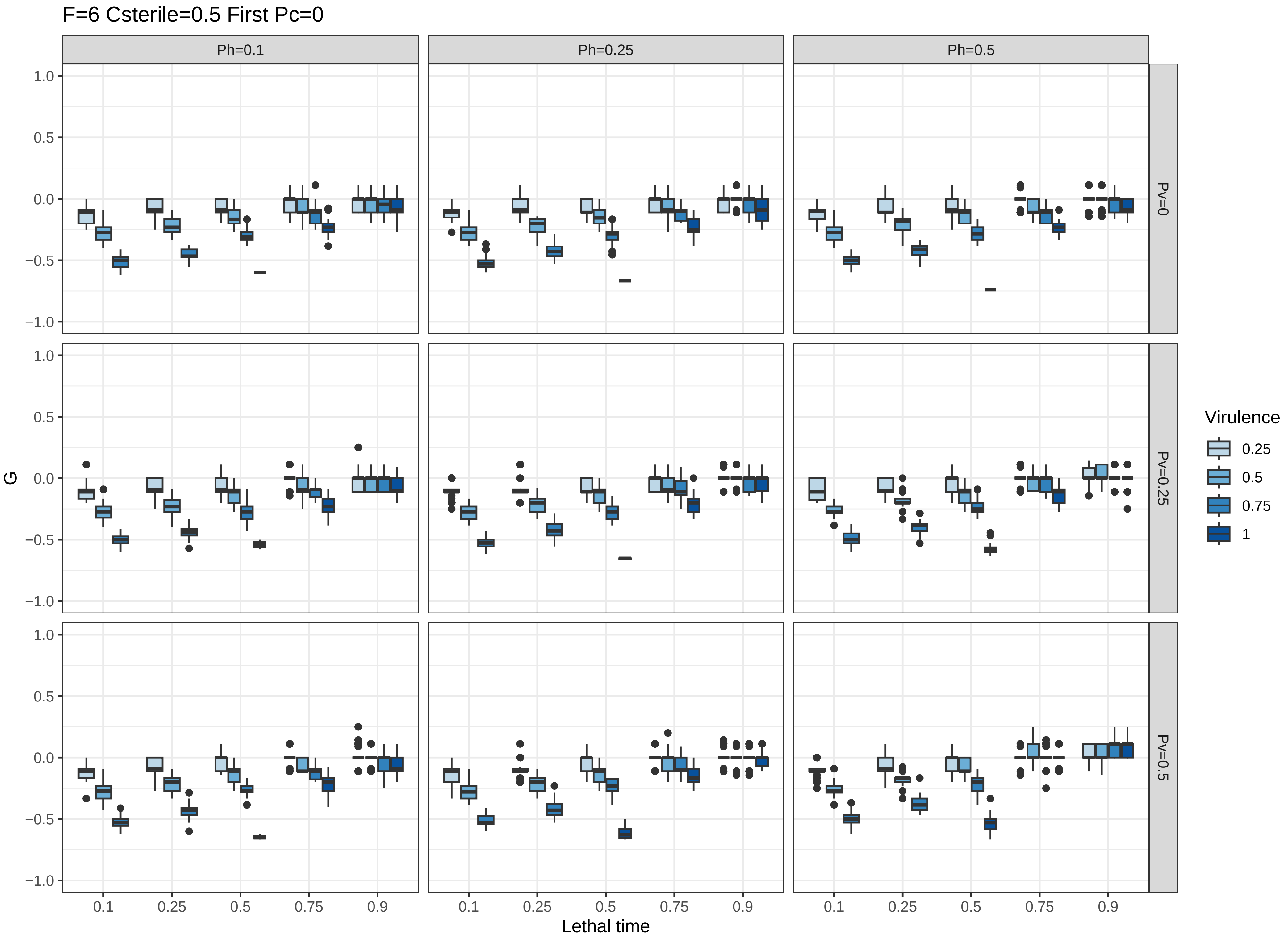

F=6 Csterile=0.5 First Pc=0
Ph=0.1
Ph=0.25
Ph=0.5
Pv=0
Pv=0.25
Pv=0.5
G
1.0
0.5
0.0
−0.5
−1.0
0.1
0.25
0.5
0.75
0.9
Lethal time
Virulence
0.25
0.5
0.75
1

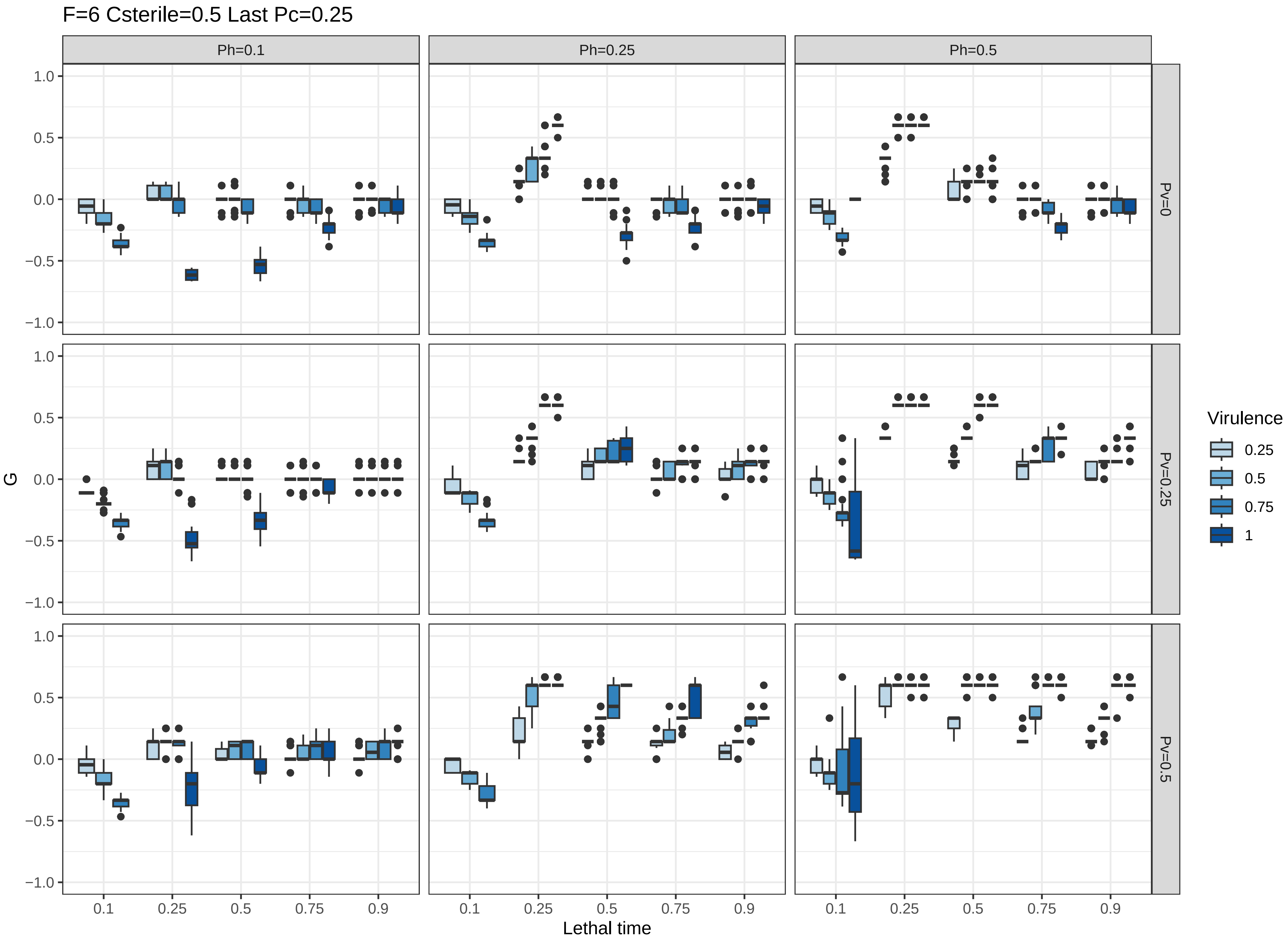

F=6 Csterile=0.5 Last Pc=0.25
Ph=0.1
Ph=0.25
Ph=0.5
Pv=0
Pv=0.25
Pv=0.5
G
1.0
0.5
0.0
−0.5
−1.0
0.1
0.25
0.5
0.75
0.9
Lethal time
Virulence
0.25
0.5
0.75
1

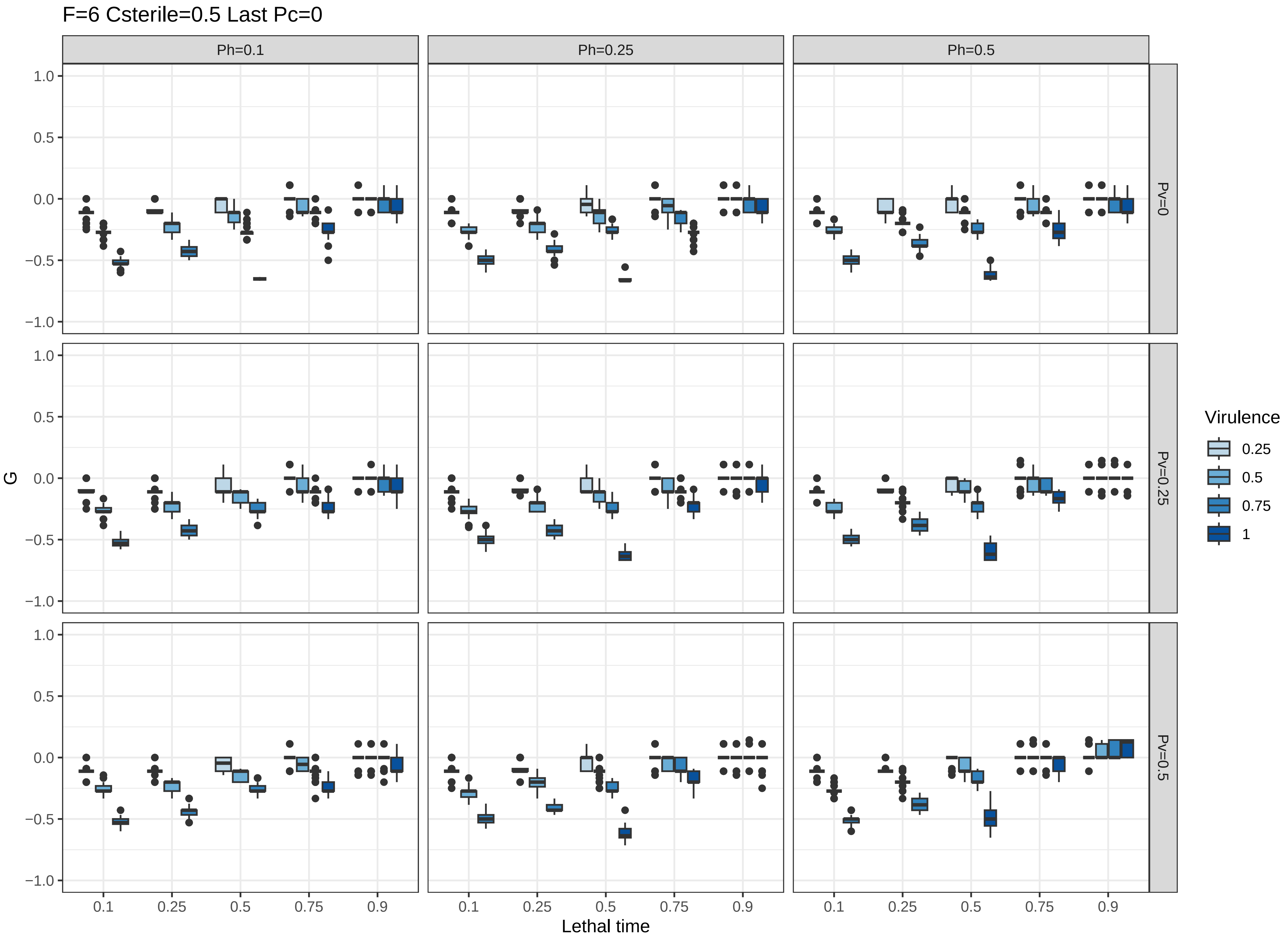

F=6 Csterile=0.5 Last Pc=0
Ph=0.1
Ph=0.25
Ph=0.5
Pv=0
Pv=0.25
Pv=0.5
G
1.0
0.5
0.0
−0.5
−1.0
0.1
0.25
0.5
0.75
0.9
Lethal time
Virulence
0.25
0.5
0.75
1

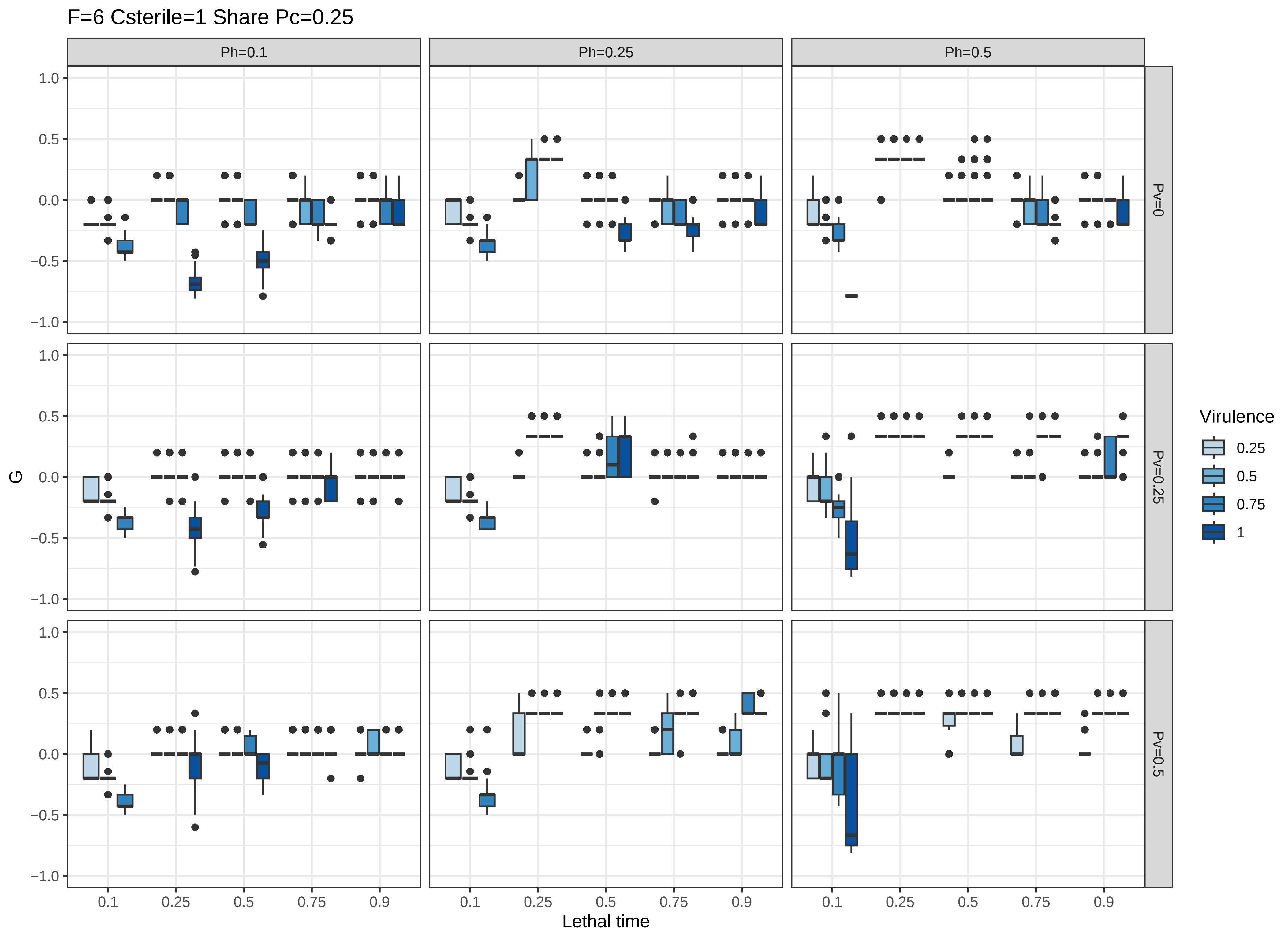

F=6 Csterile=1 Share Pc=0.25
Ph=0.1
Ph=0.25
Ph=0.5
Pv=0
Pv=0.25
Pv=0.5
G
1.0
0.5
0.0
−0.5
−1.0
Lethal time
0.1
0.25
0.5
0.75
0.9
Virulence
0.25
0.5
0.75
1

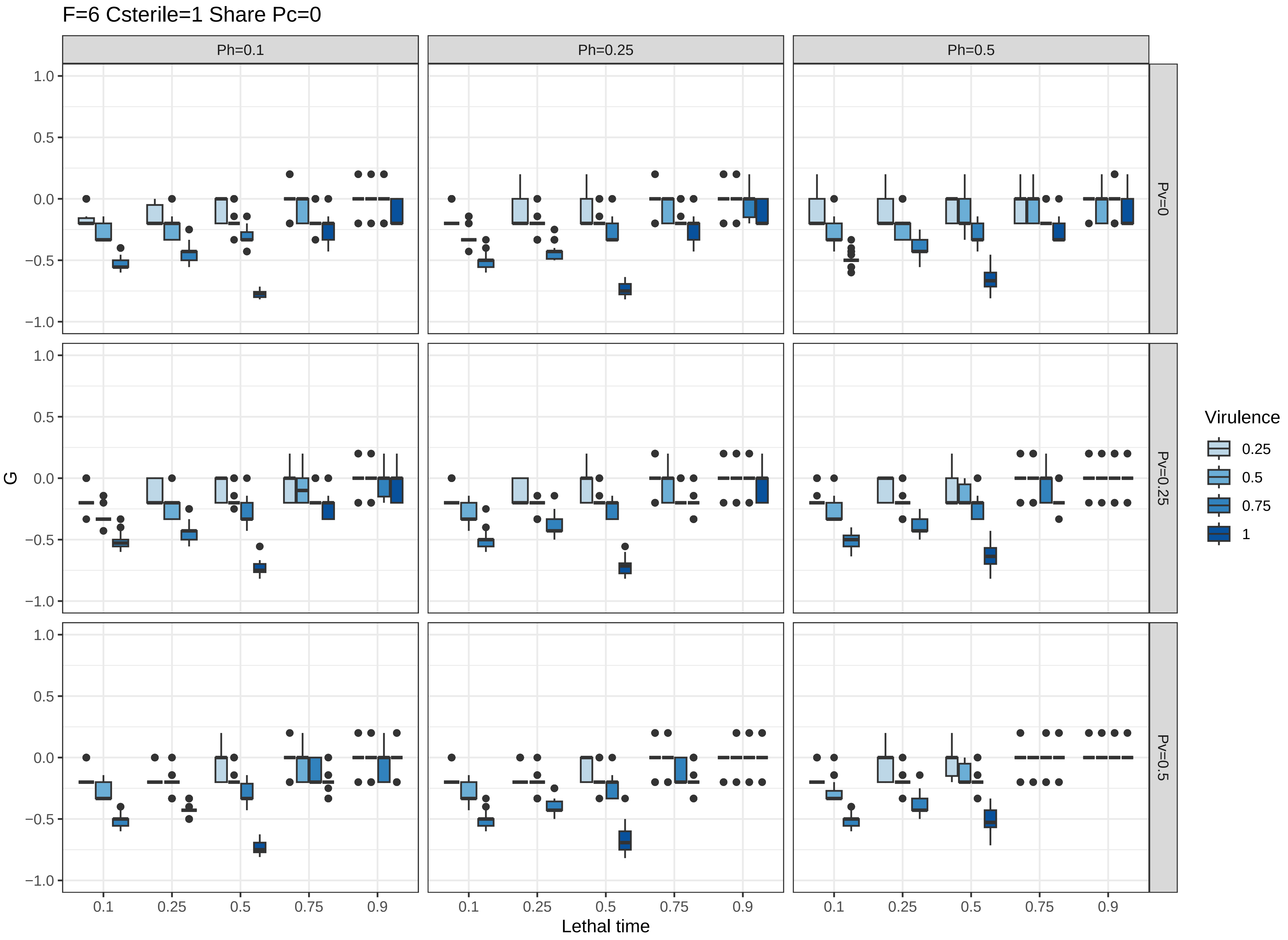

F=6 Csterile=1 Share Pc=0
Ph=0.1
Ph=0.25
Ph=0.5
Pv=0
Pv=0.25
Pv=0.5
G
1.0
0.5
0.0
−0.5
−1.0
0.1
0.25
0.5
0.75
0.9
Lethal time
Virulence
0.25
0.5
0.75
1

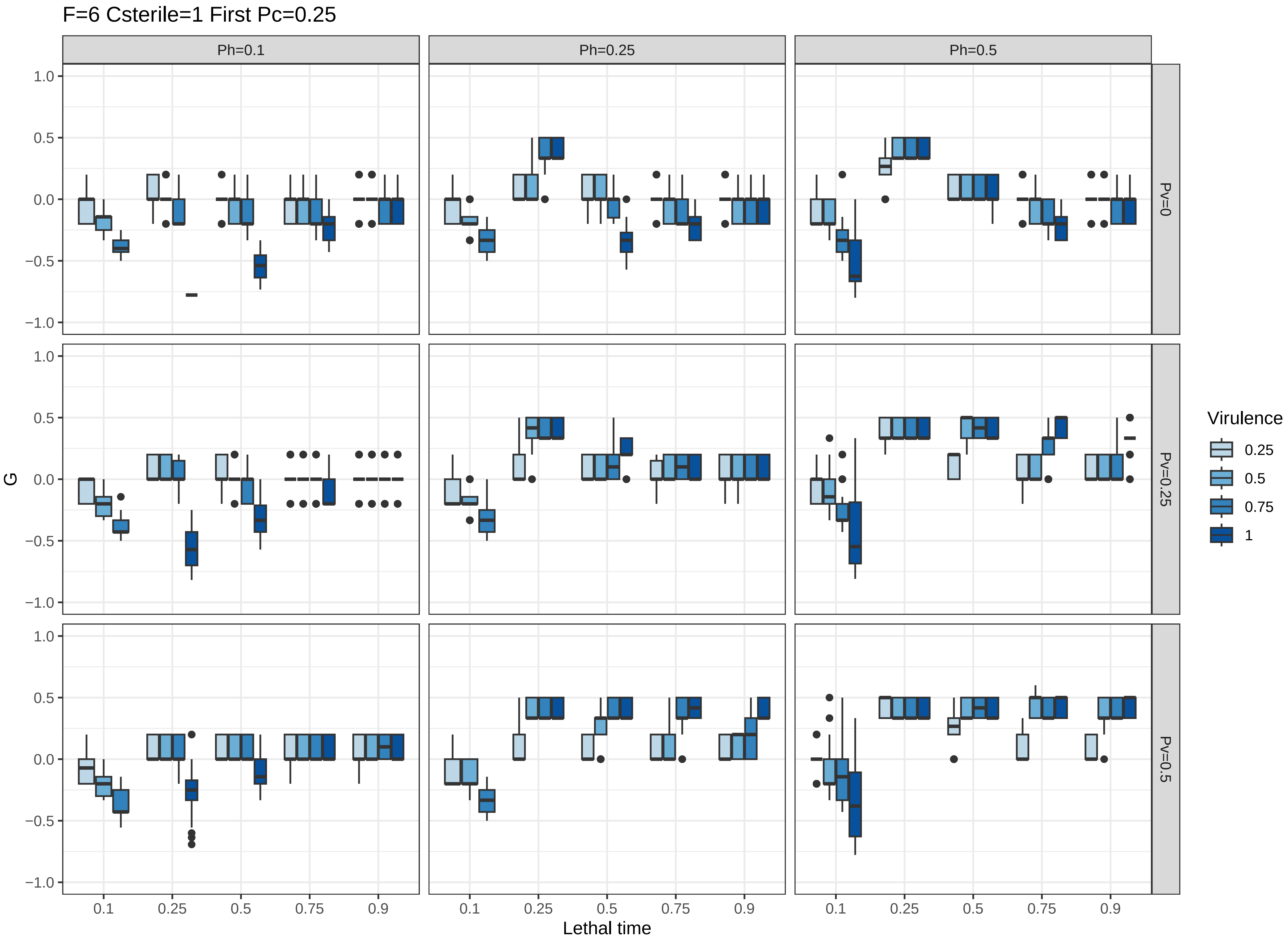

F=6 Csterile=1 First Pc=0.25
Ph=0.1
Ph=0.25
Ph=0.5
Pv=0
Pv=0.25
Pv=0.5
G
1.0
0.5
0.0
−0.5
−1.0
Lethal time
0.1
0.25
0.5
0.75
0.9
Virulence
0.25
0.5
0.75
1

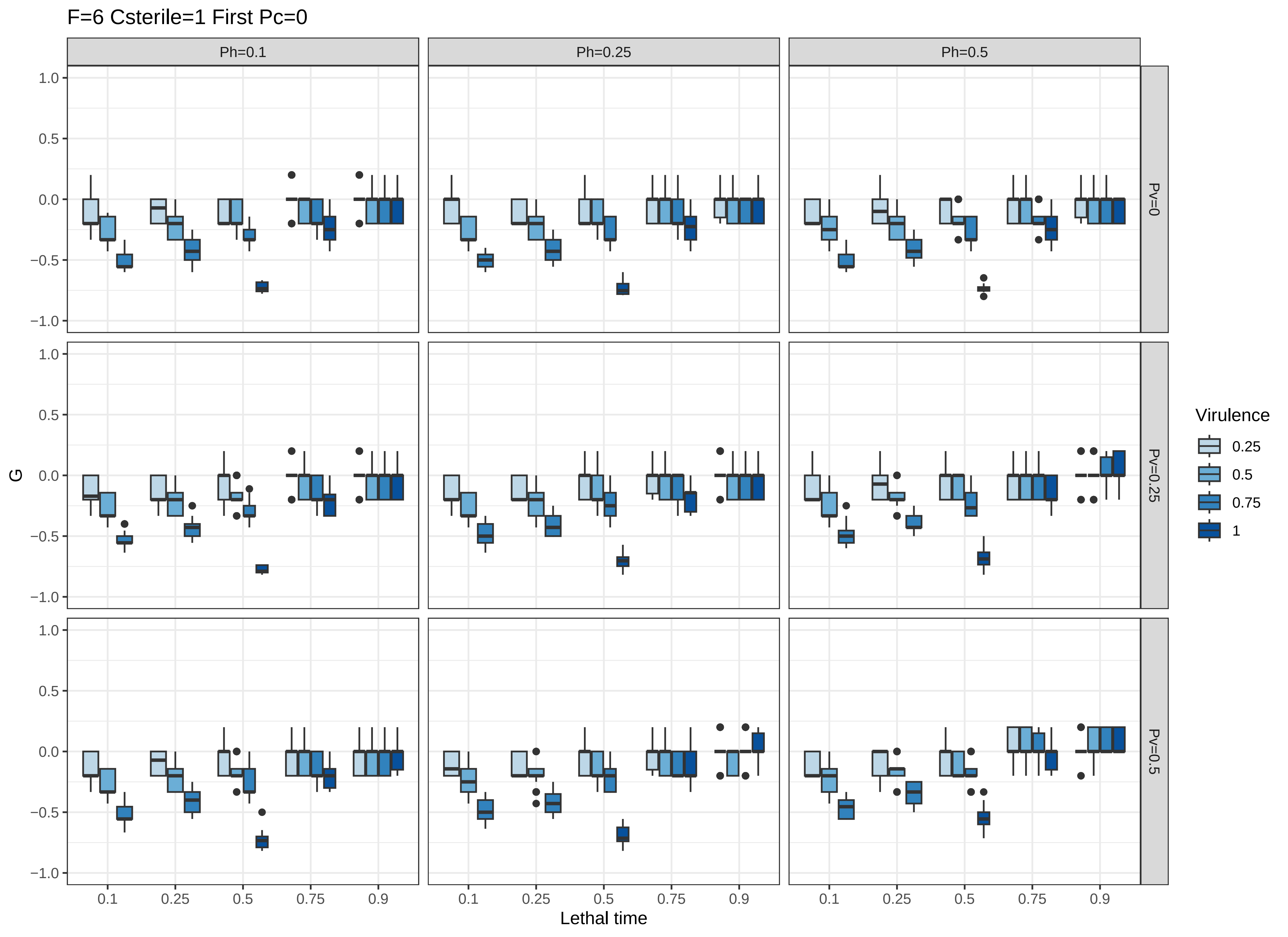

F=6 Csterile=1 First Pc=0
Ph=0.1
Ph=0.25
Ph=0.5
Pv=0
Pv=0.25
Pv=0.5
G
1.0
0.5
0.0
−0.5
−1.0
0.1
0.25
0.5
0.75
0.9
Lethal time
Virulence
0.25
0.5
0.75
1

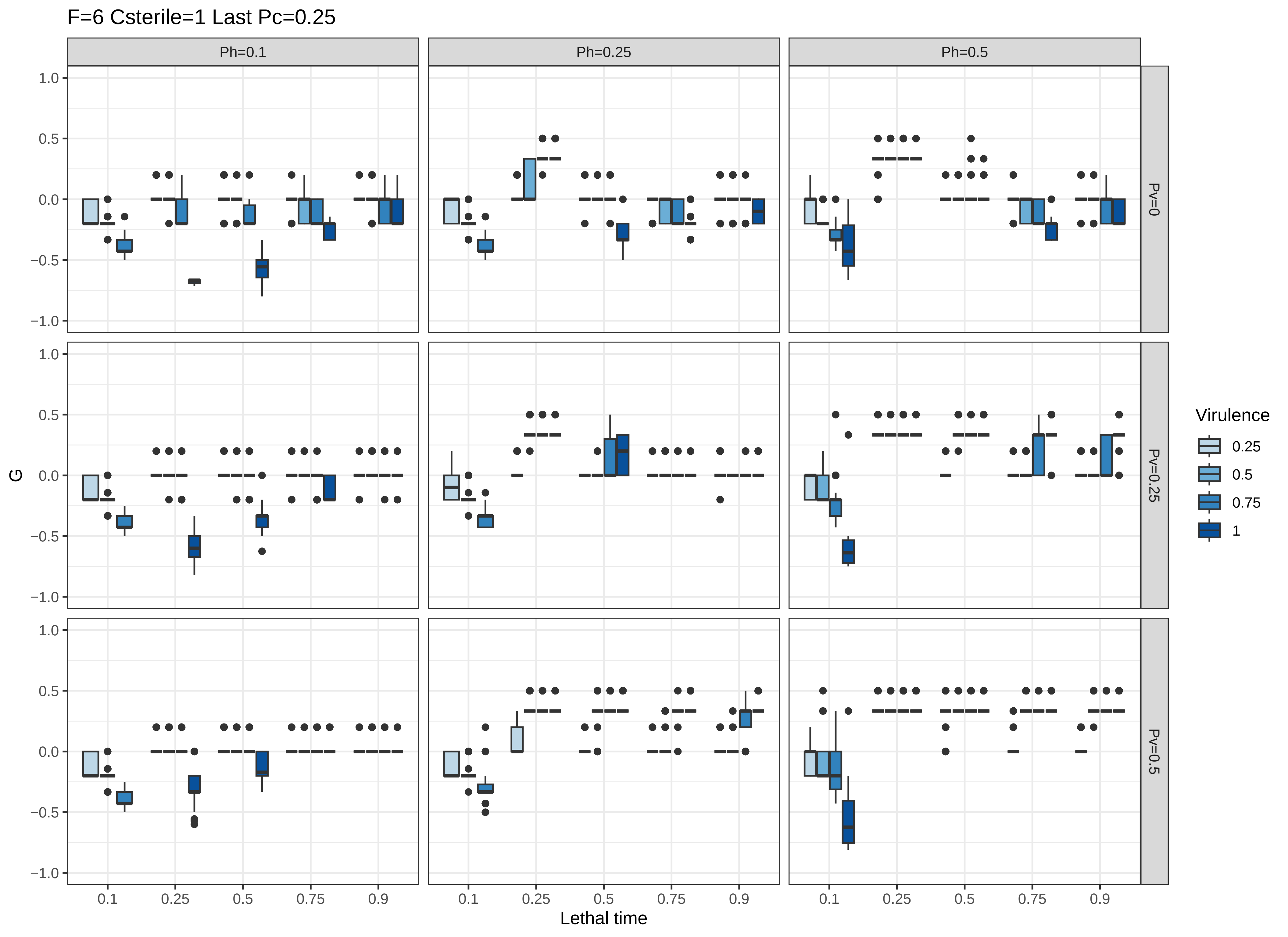
F=6 Csterile=1 Last Pc=0.25
Ph=0.1
Ph=0.25
Ph=0.5
Pv=0
Pv=0.25
Pv=0.5
G
Lethal time
1.0
0.5
0.0
−0.5
−1.0
0.1
0.25
0.5
0.75
0.9
Virulence
0.25
0.5
0.75
1

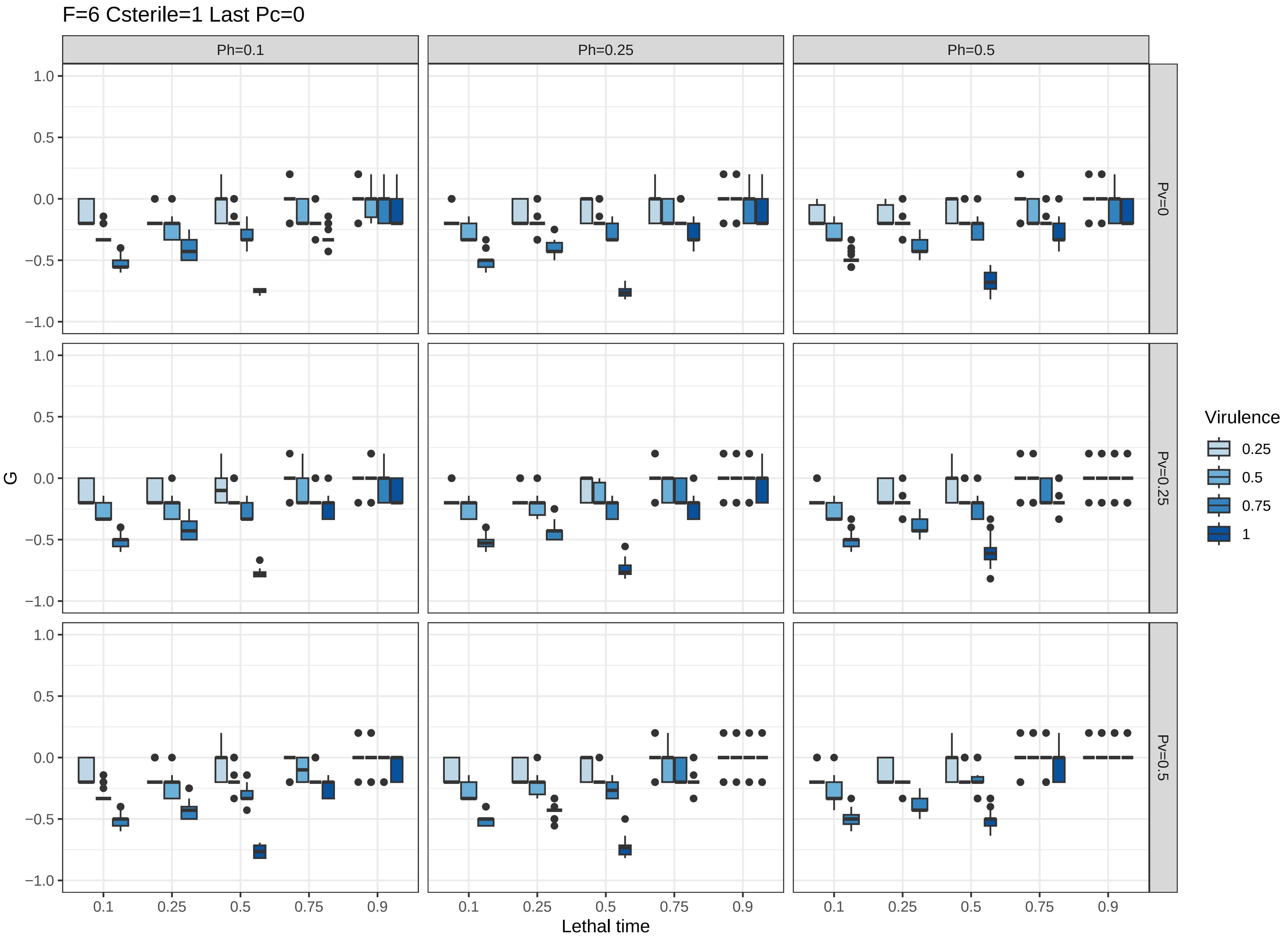
F=6 Csterile=1 Last Pc=0
Ph=0.1
Ph=0.25
Ph=0.5
Pv=0
Pv=0.25
Pv=0.5
G
1.0
0.5
0.0
−0.5
−1.0
0.1
0.25
0.5
0.75
0.9
Lethal time
Virulence
0.25
0.5
0.75
1

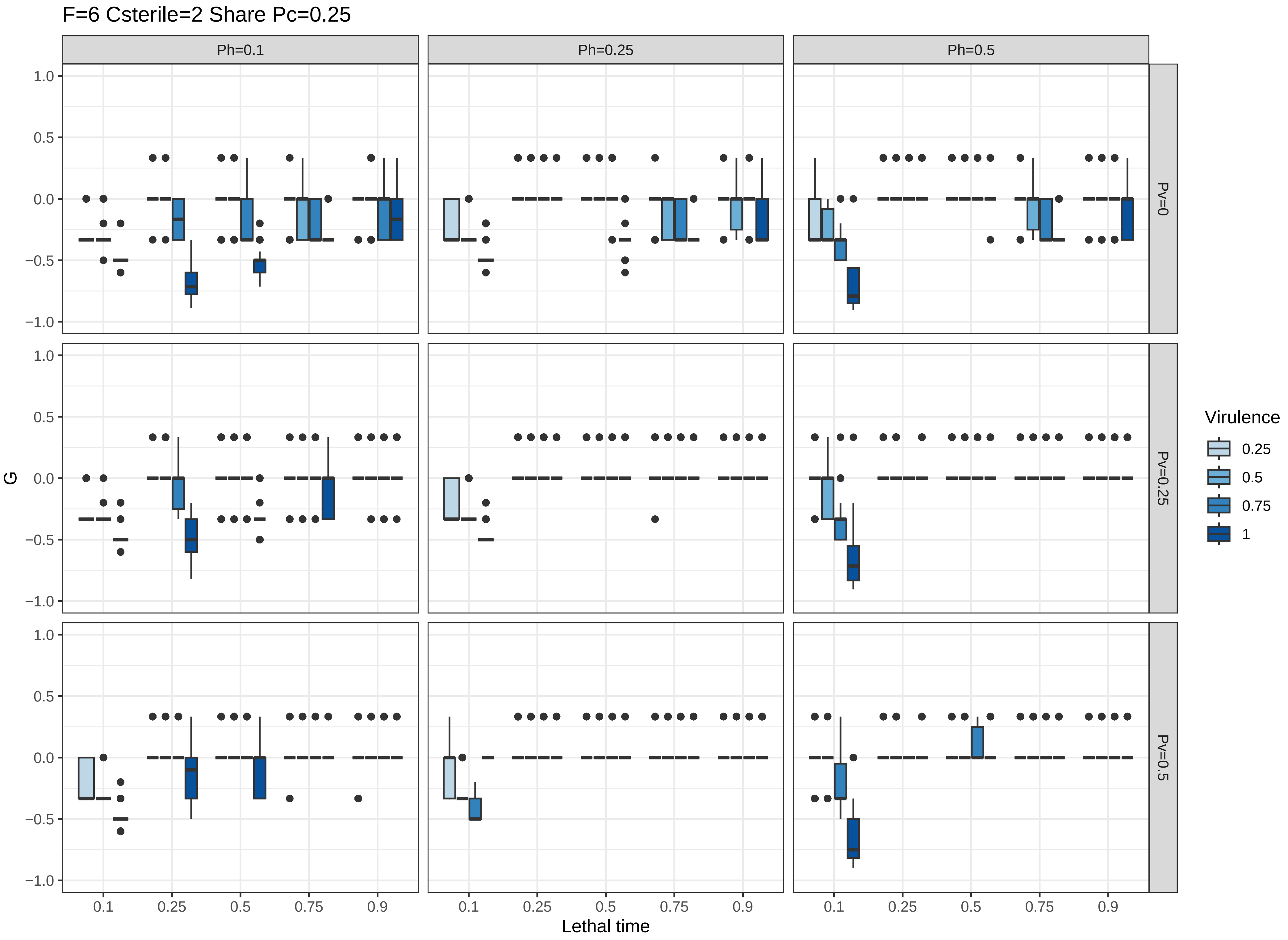

F=6 Csterile=2 Share Pc=0.25
Ph=0.1
Ph=0.25
Ph=0.5
Pv=0
Pv=0.25
Pv=0.5
G
Lethal time
0.1
0.25
0.5
0.75
0.9
1.0
0.5
0.0
−0.5
−1.0
Virulence
0.25
0.5
0.75
1

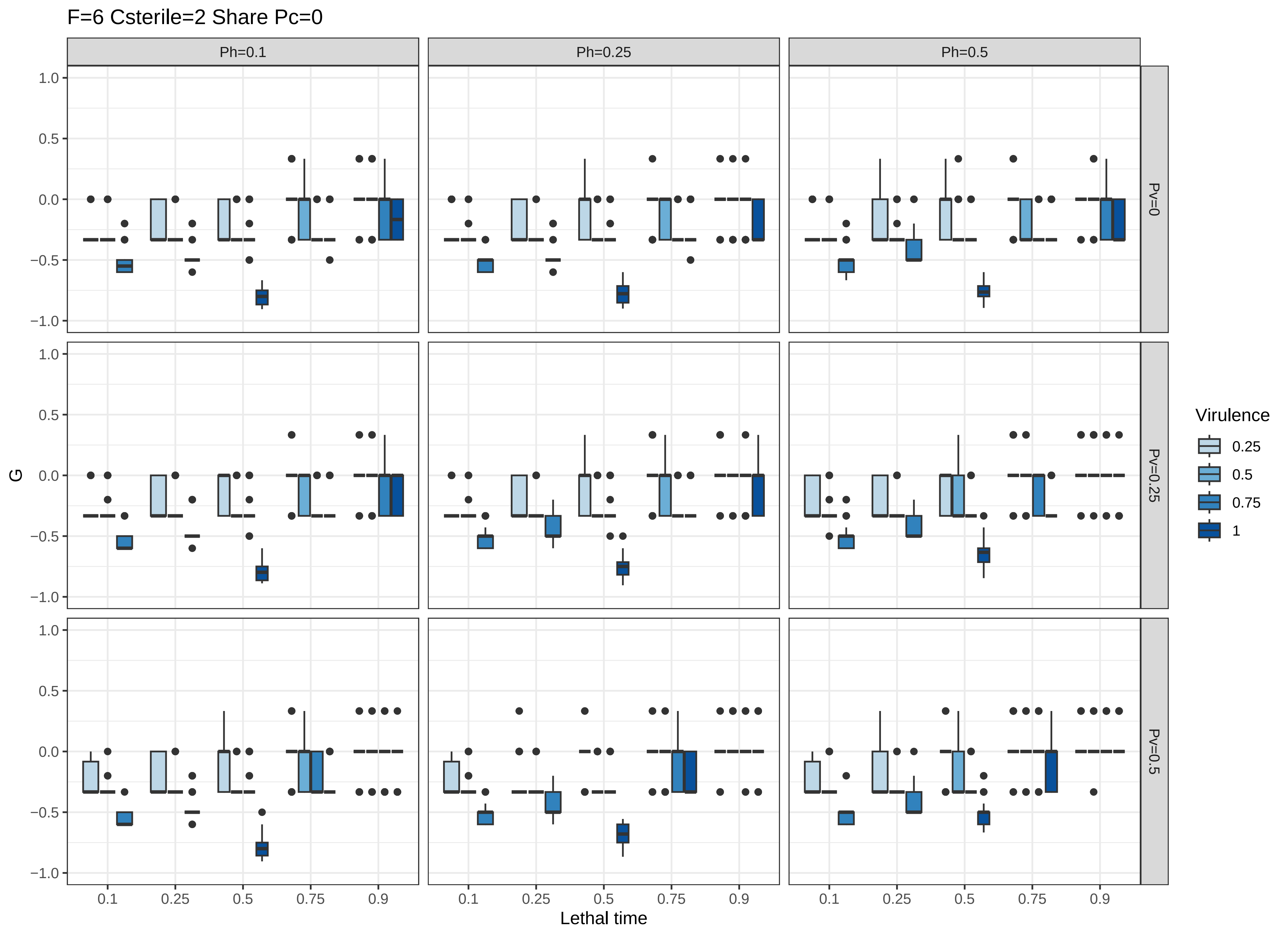

F=6 Csterile=2 Share Pc=0
Ph=0.1
Ph=0.25
Ph=0.5
Pv=0
Pv=0.25
Pv=0.5
G
1.0
0.5
0.0
−0.5
−1.0
0.1
0.25
0.5
0.75
0.9
Lethal time
Virulence
0.25
0.5
0.75
1

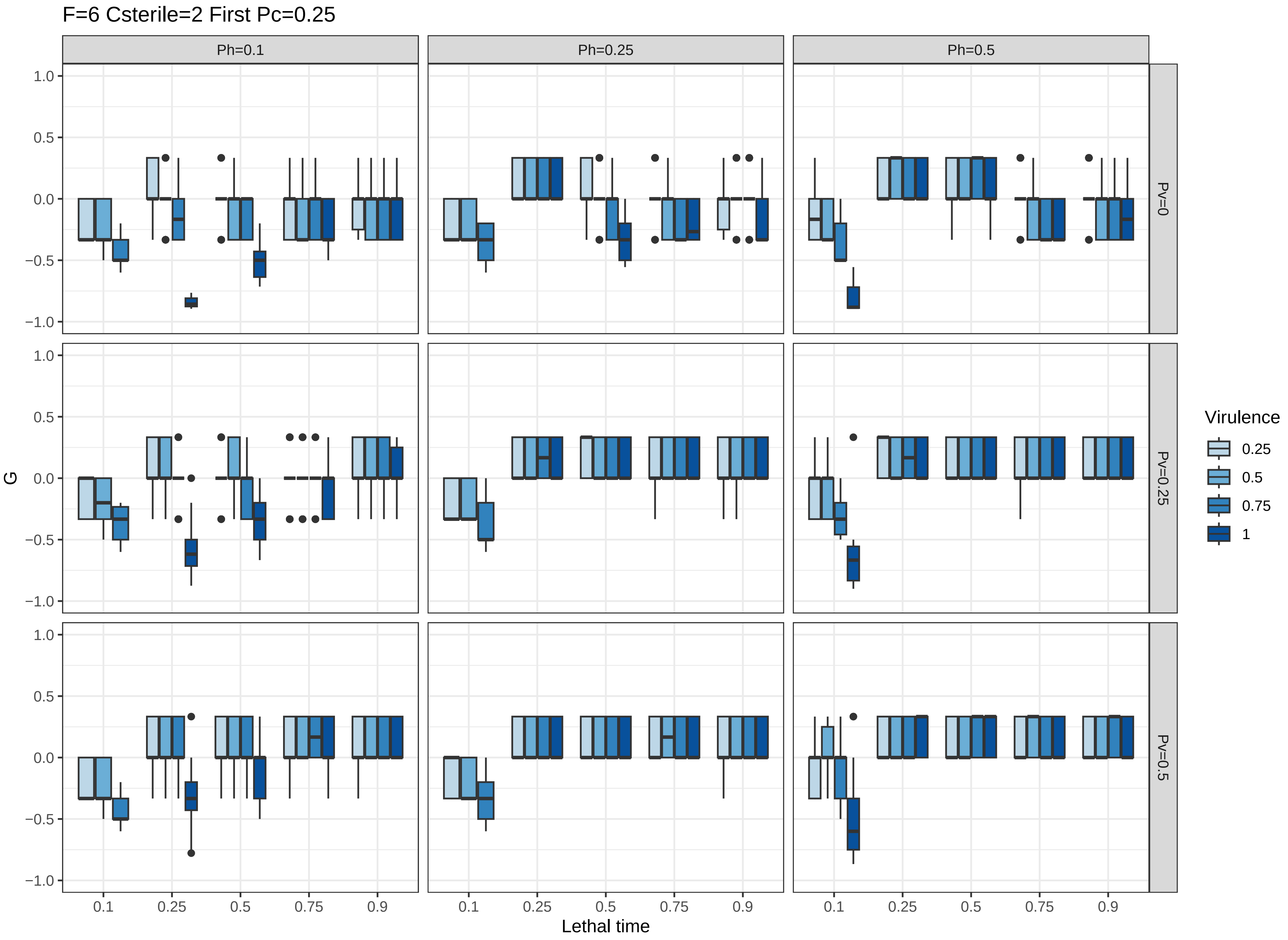

F=6 Csterile=2 First Pc=0.25
Ph=0.1
Ph=0.25
Ph=0.5
Pv=0
Pv=0.25
Pv=0.5
1.0
0.5
0.0
−0.5
−1.0
G
0.1
0.25
0.5
0.75
0.9
Lethal time
Virulence
0.25
0.5
0.75
1

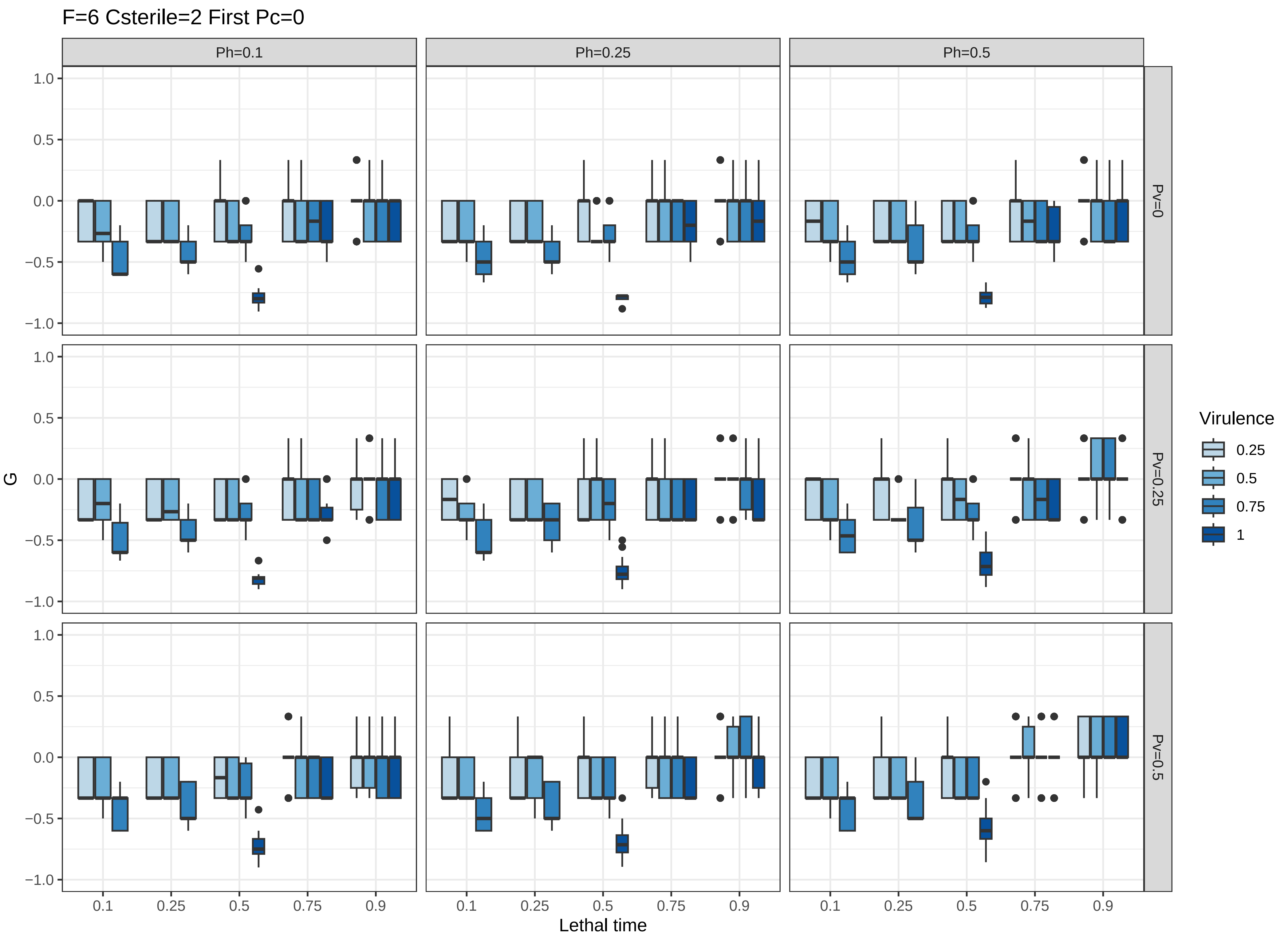

F=6 Csterile=2 First Pc=0
Ph=0.1
Ph=0.25
Ph=0.5
Pv=0
Pv=0.25
Pv=0.5
G
1.0
0.5
0.0
−0.5
−1.0
0.1
0.25
0.5
0.75
0.9
Lethal time
Virulence
0.25
0.5
0.75
1

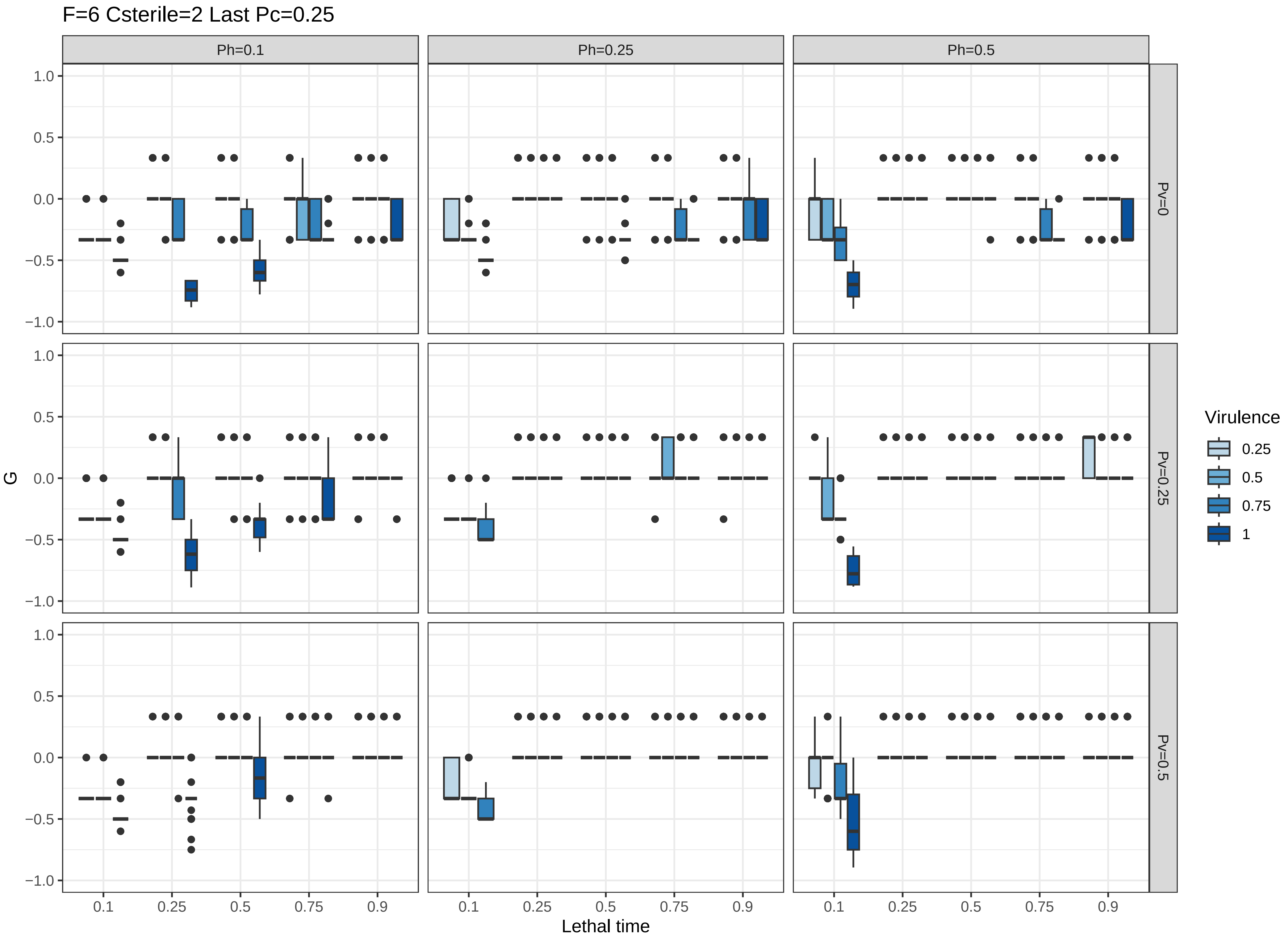
F=6 Csterile=2 Last Pc=0.25
Ph=0.1
Ph=0.25
Ph=0.5
Pv=0
Pv=0.25
Pv=0.5
G
Lethal time
Virulence
0.25
0.5
0.75
1
1.0
0.5
0.0
−0.5
−1.0
0.1
0.25
0.5
0.75
0.9

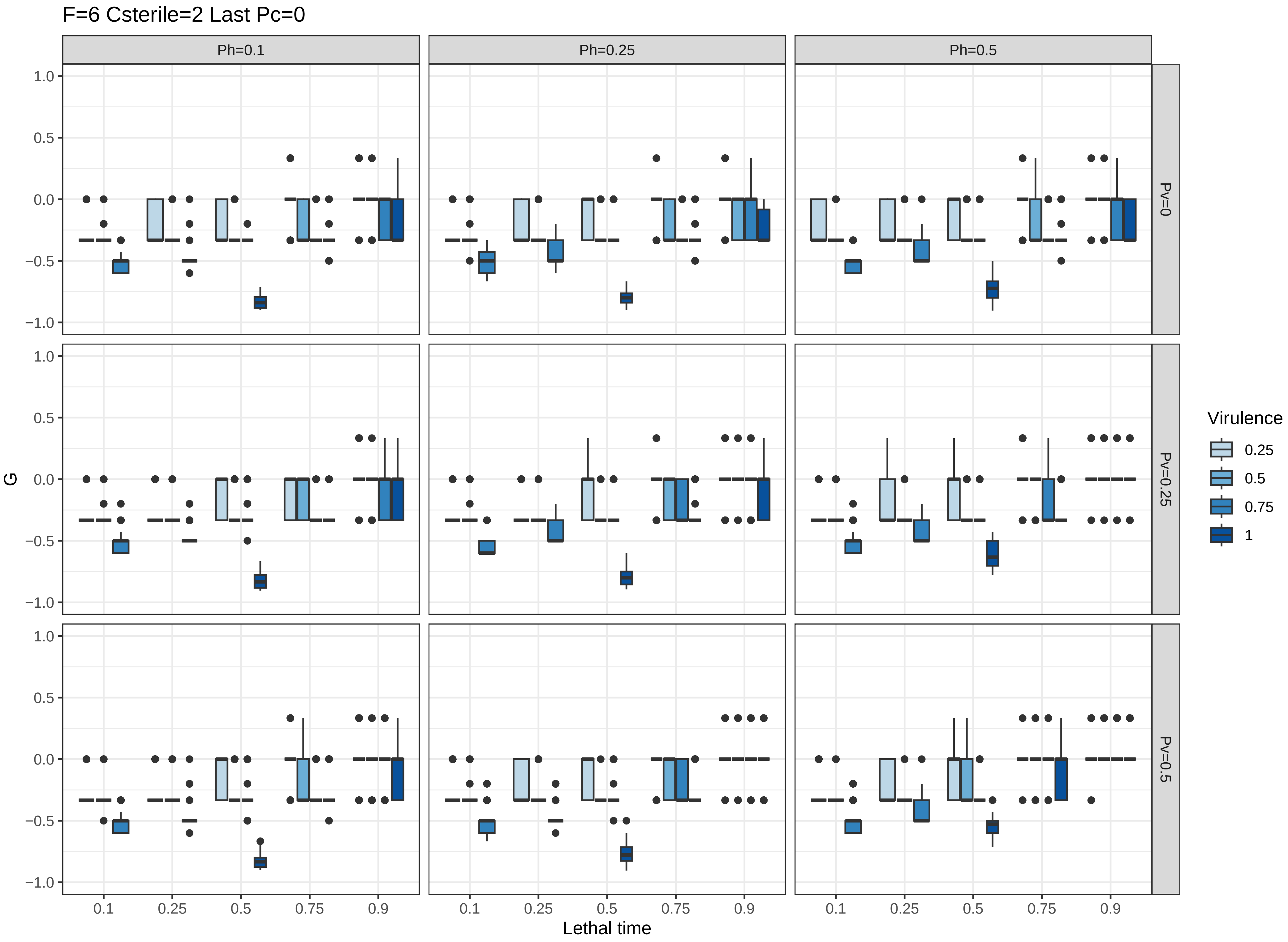

F=6 Csterile=2 Last Pc=0
Ph=0.1
Ph=0.25
Ph=0.5
Pv=0
Pv=0.25
Pv=0.5
G
Lethal time
Virulence
0.25
0.5
0.75
1

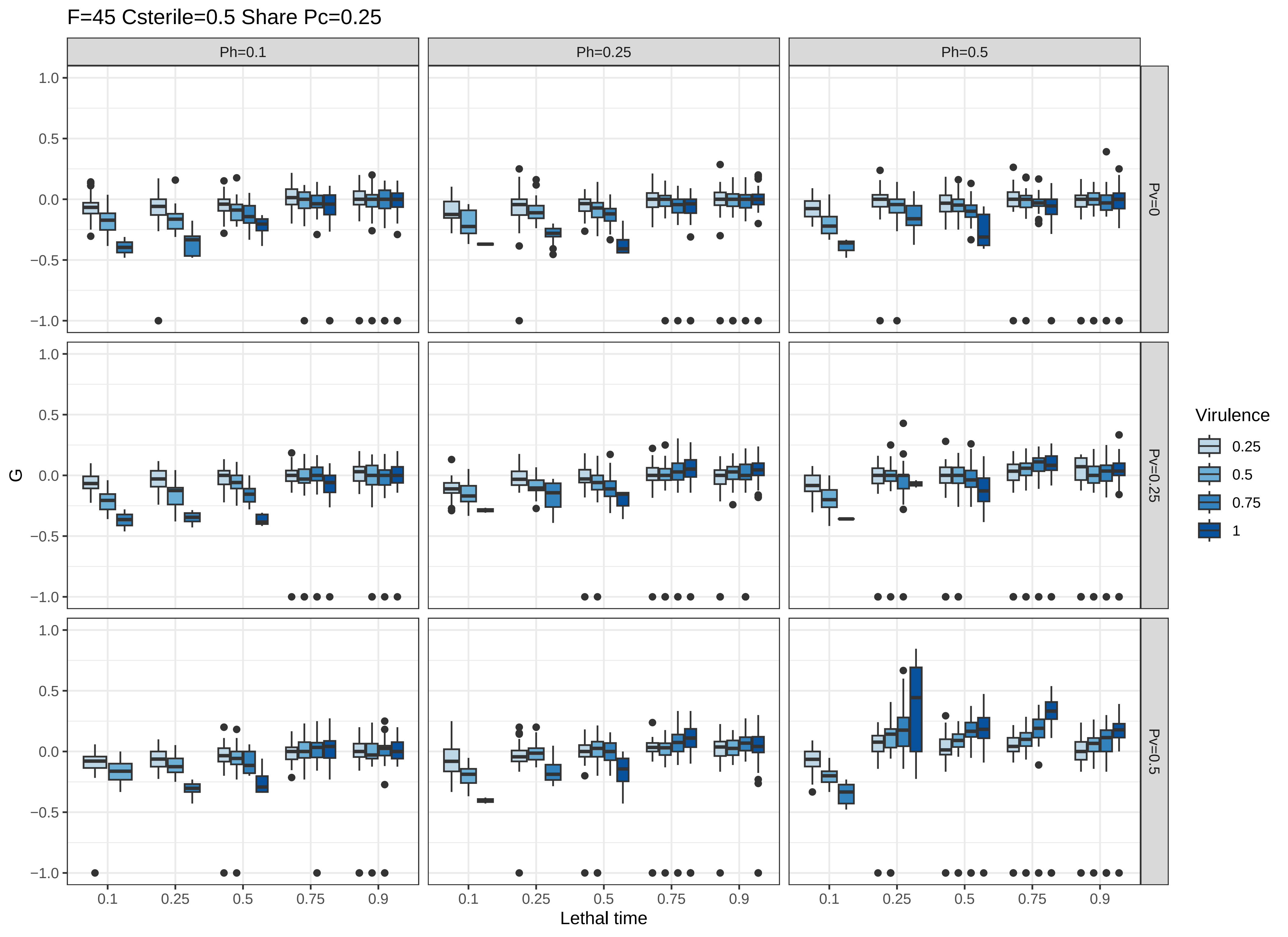
F=45 Csterile=0.5 Share Pc=0.25
Ph=0.1
Ph=0.25
Ph=0.5
Pv=0
Pv=0.25
Pv=0.5
1.0
0.5
0.0
−0.5
−1.0
G
0.1
0.25
0.5
0.75
0.9
Lethal time
Virulence
0.25
0.5
0.75
1

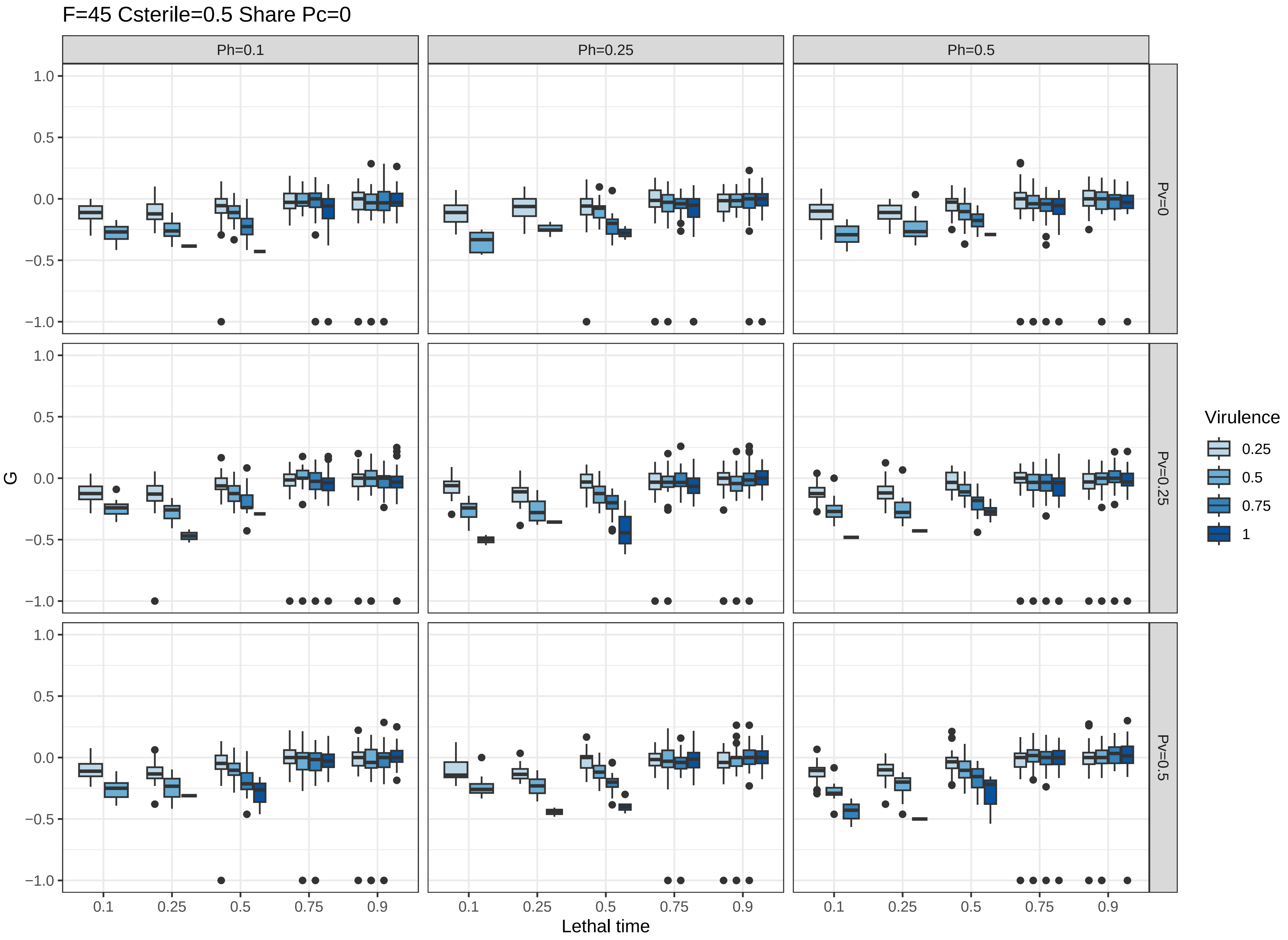

F=45 Csterile=0.5 Share Pc=0
Ph=0.1
Ph=0.25
Ph=0.5
Pv=0
Pv=0.25
Pv=0.5
G
1.0
0.5
0.0
−0.5
−1.0
0.1
0.25
0.5
0.75
0.9
Lethal time
Virulence
0.25
0.5
0.75
1

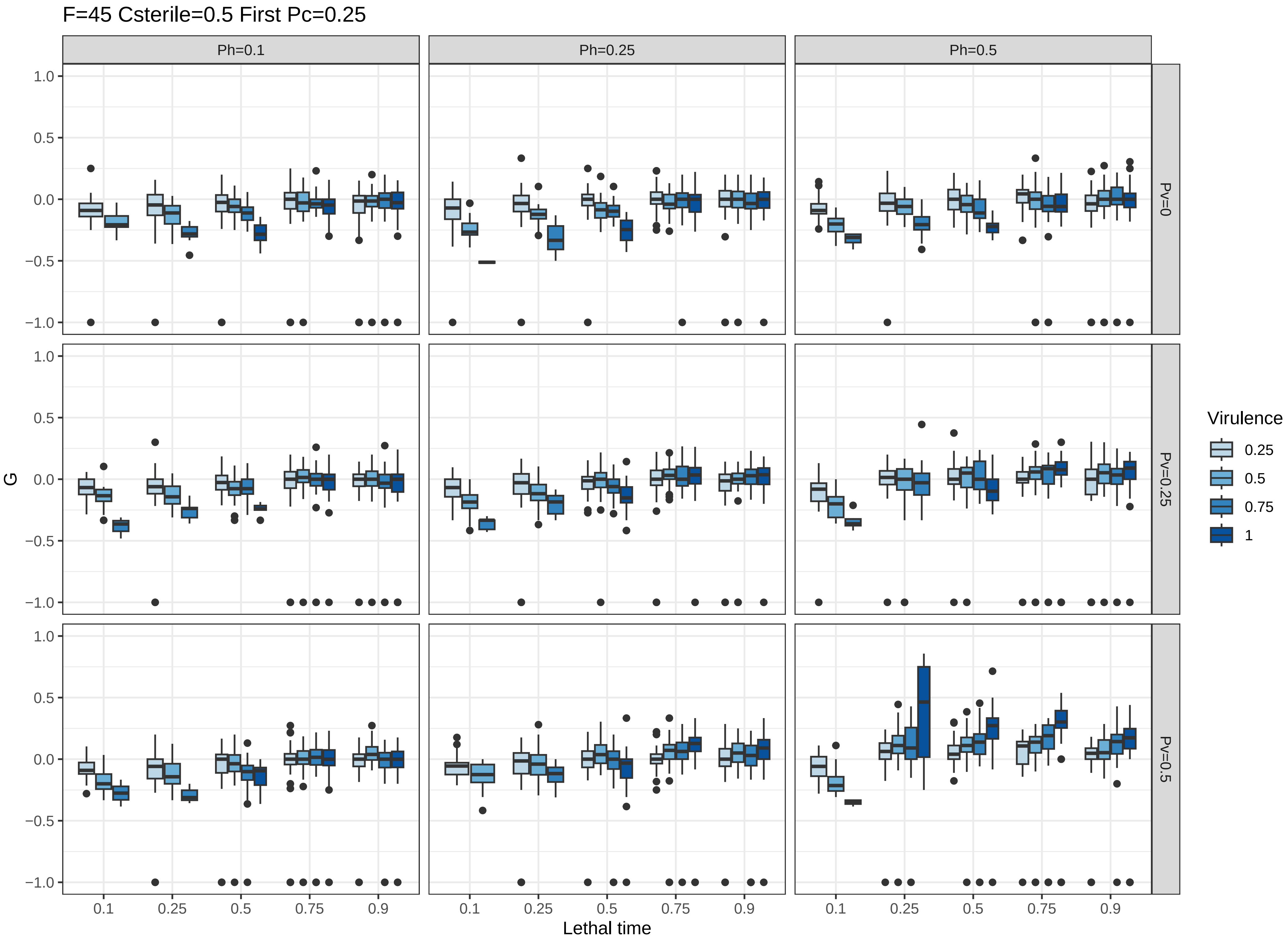

F=45 Csterile=0.5 First Pc=0.25
Ph=0.1
Ph=0.25
Ph=0.5
Pv=0
Pv=0.25
Pv=0.5
G
1.0
0.5
0.0
−0.5
−1.0
0.1
0.25
0.5
0.75
0.9
Lethal time
Virulence
0.25
0.5
0.75
1

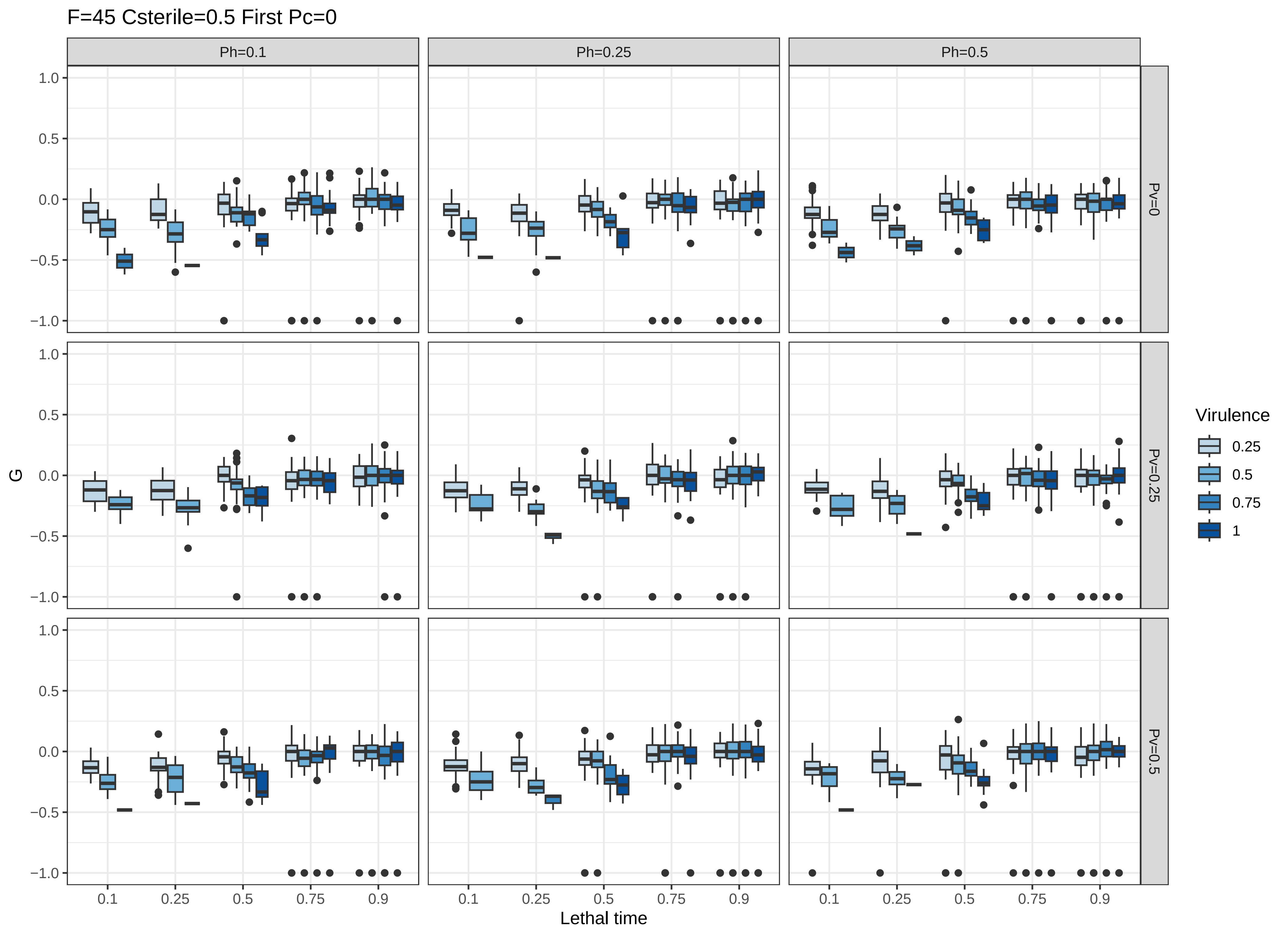

F=45 Csterile=0.5 First Pc=0
Ph=0.1
Ph=0.25
Ph=0.5
Pv=0
Pv=0.25
Pv=0.5
1.0
0.5
0.0
−0.5
−1.0
G
0.1
0.25
0.5
0.75
0.9
Lethal time
Virulence
0.25
0.5
0.75
1

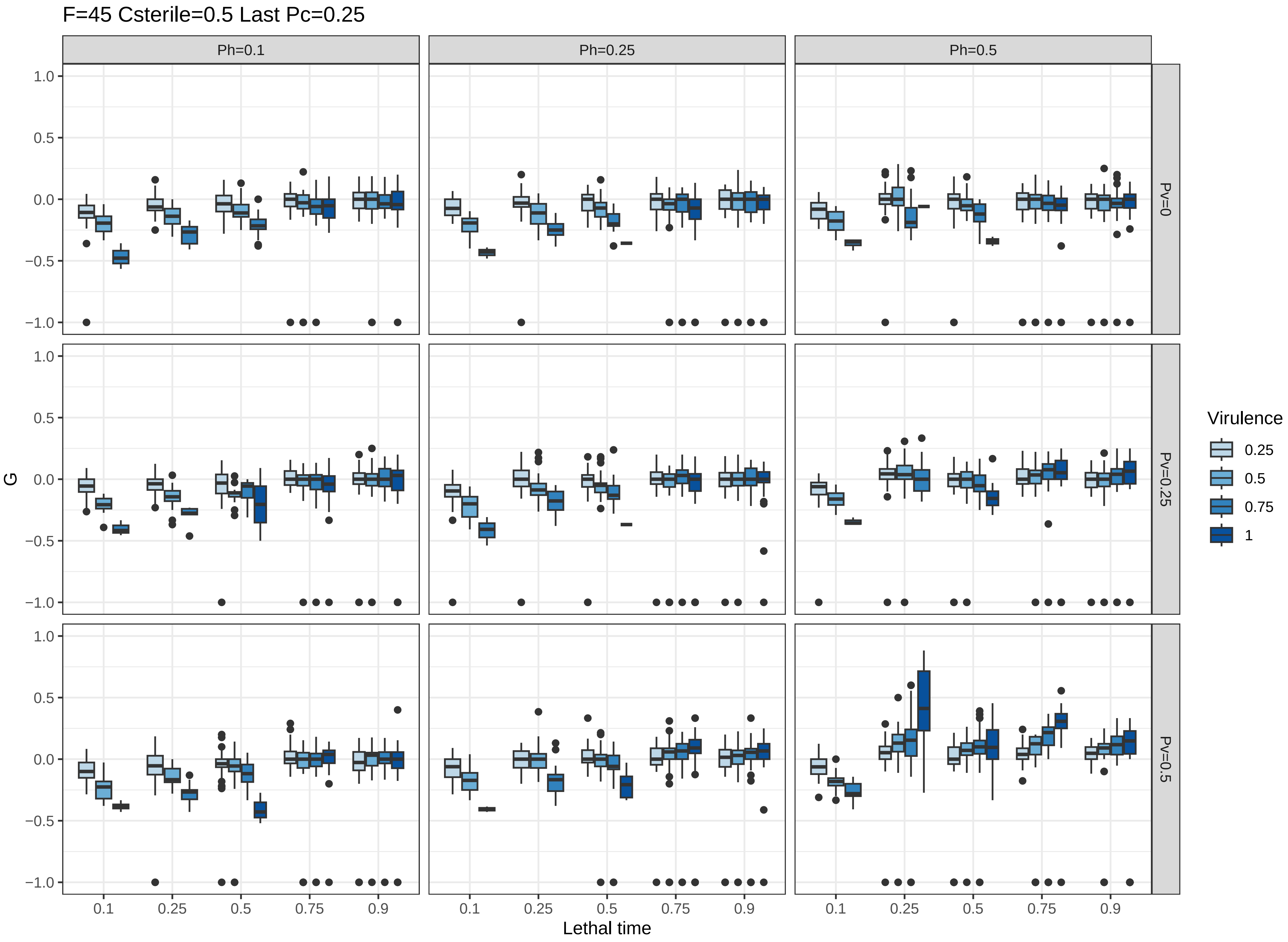

F=45 Csterile=0.5 Last Pc=0.25
Ph=0.1
Ph=0.25
Ph=0.5
Pv=0
Pv=0.25
Pv=0.5
G
1.0
0.5
0.0
−0.5
−1.0
0.1
0.25
0.5
0.75
0.9
Lethal time
Virulence
0.25
0.5
0.75
1

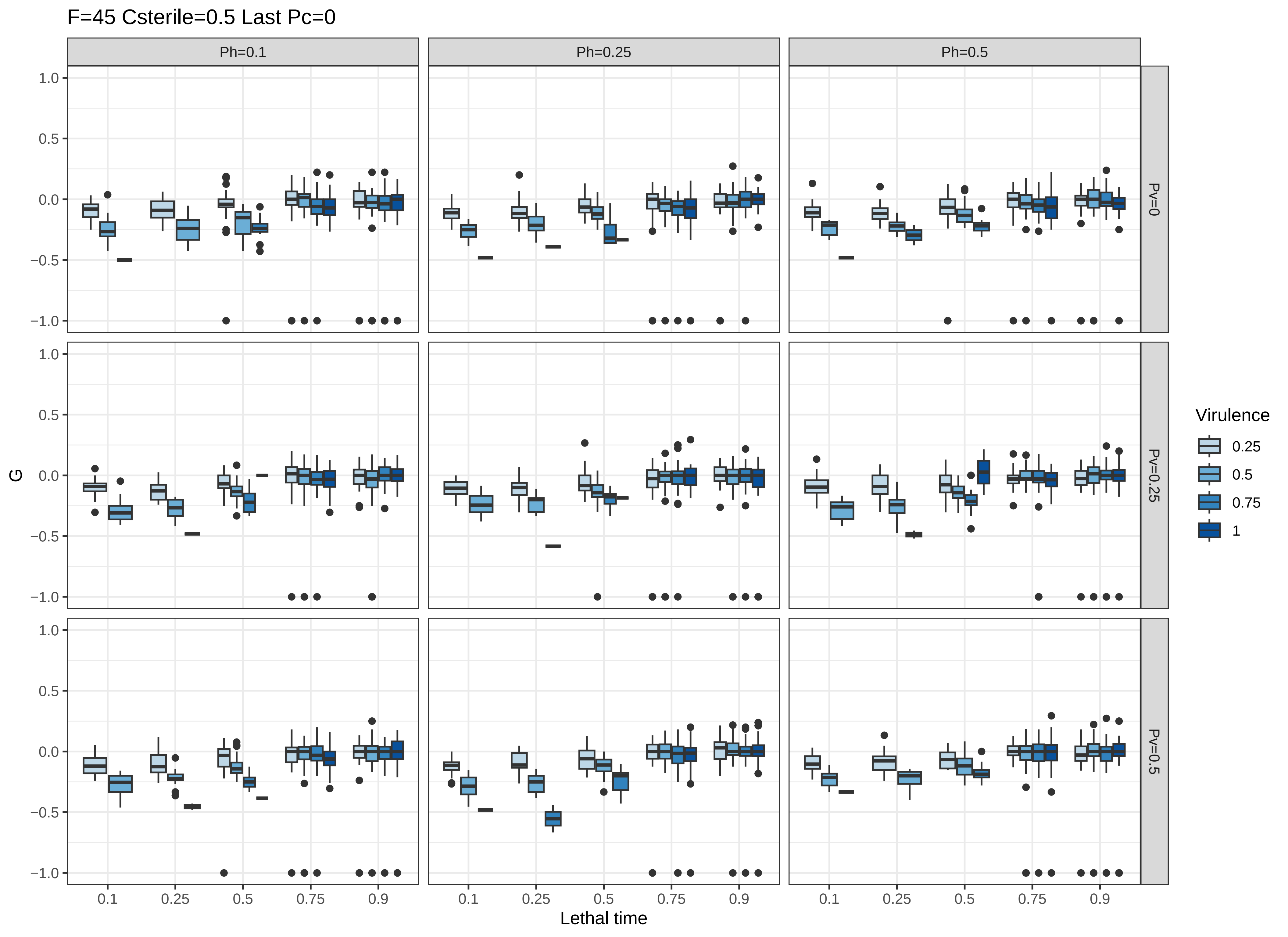

F=45 Csterile=0.5 Last Pc=0
Ph=0.1
Ph=0.25
Ph=0.5
Pv=0
Pv=0.25
Pv=0.5
G
1.0
0.5
0.0
−0.5
−1.0
0.1
0.25
0.5
0.75
0.9
Lethal time
Virulence
0.25
0.5
0.75
1

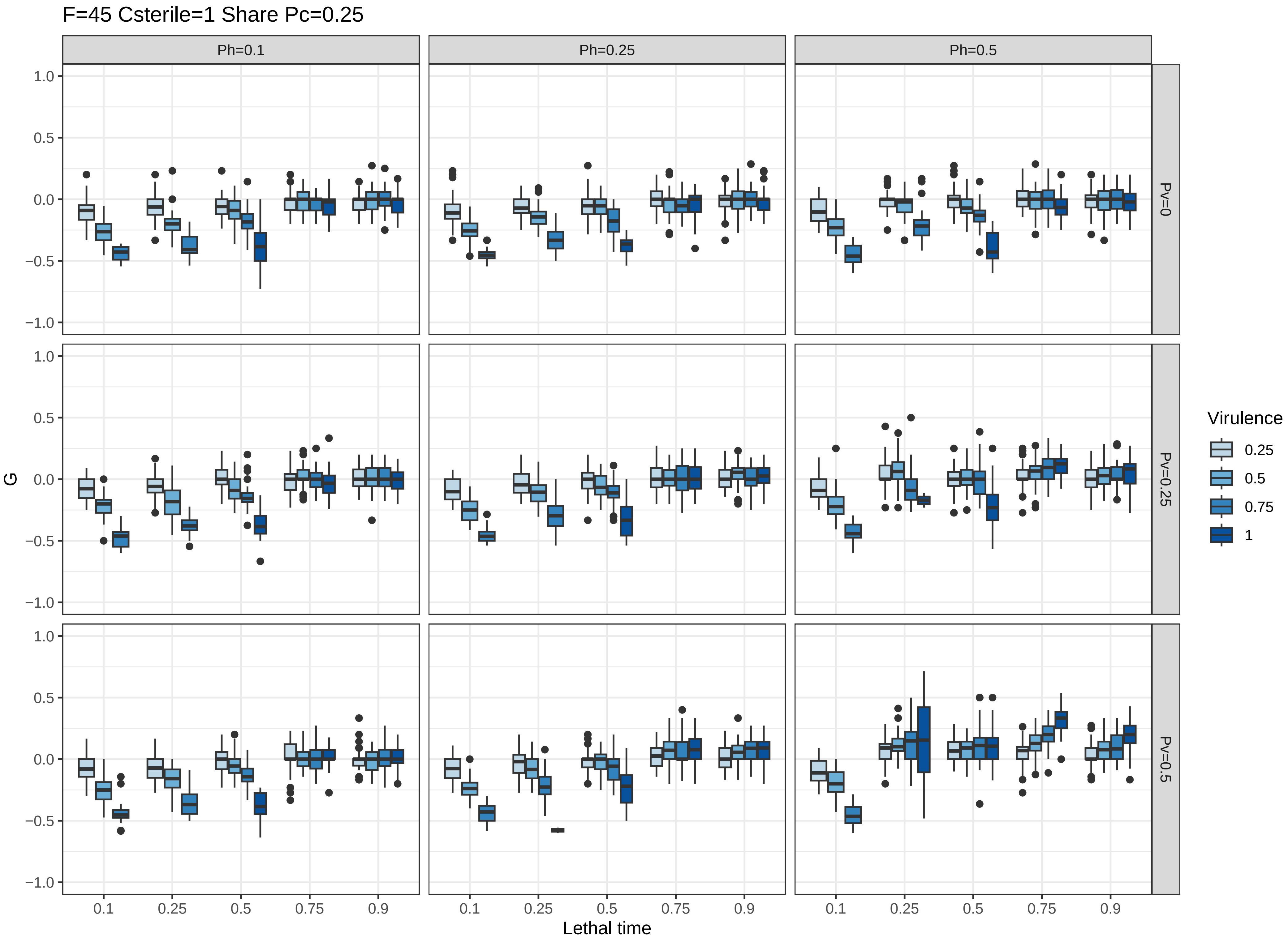
F=45 Csterile=1 Share Pc=0.25
Ph=0.1
Ph=0.25
Ph=0.5
Pv=0
Pv=0.25
Pv=0.5
G
Lethal time
Virulence
0.25
0.5
0.75
1
1.0
0.5
0.0
−0.5
−1.0
0.1
0.25
0.5
0.75
0.9

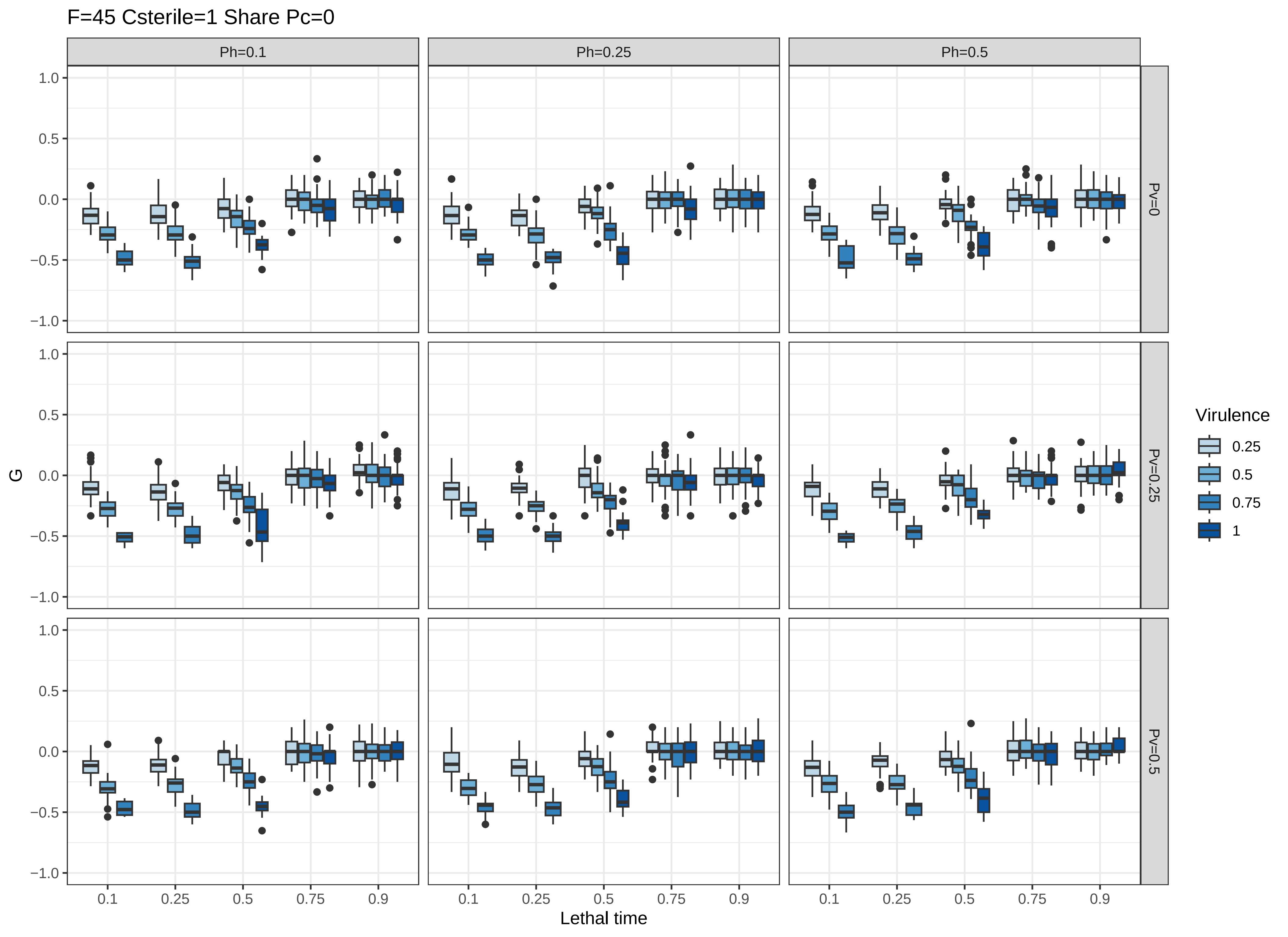

F=45 Csterile=1 Share Pc=0
Ph=0.1
Ph=0.25
Ph=0.5
Pv=0
Pv=0.25
Pv=0.5
1.0
0.5
0.0
−0.5
−1.0
G
0.1
0.25
0.5
0.75
0.9
Lethal time
Virulence
0.25
0.5
0.75
1

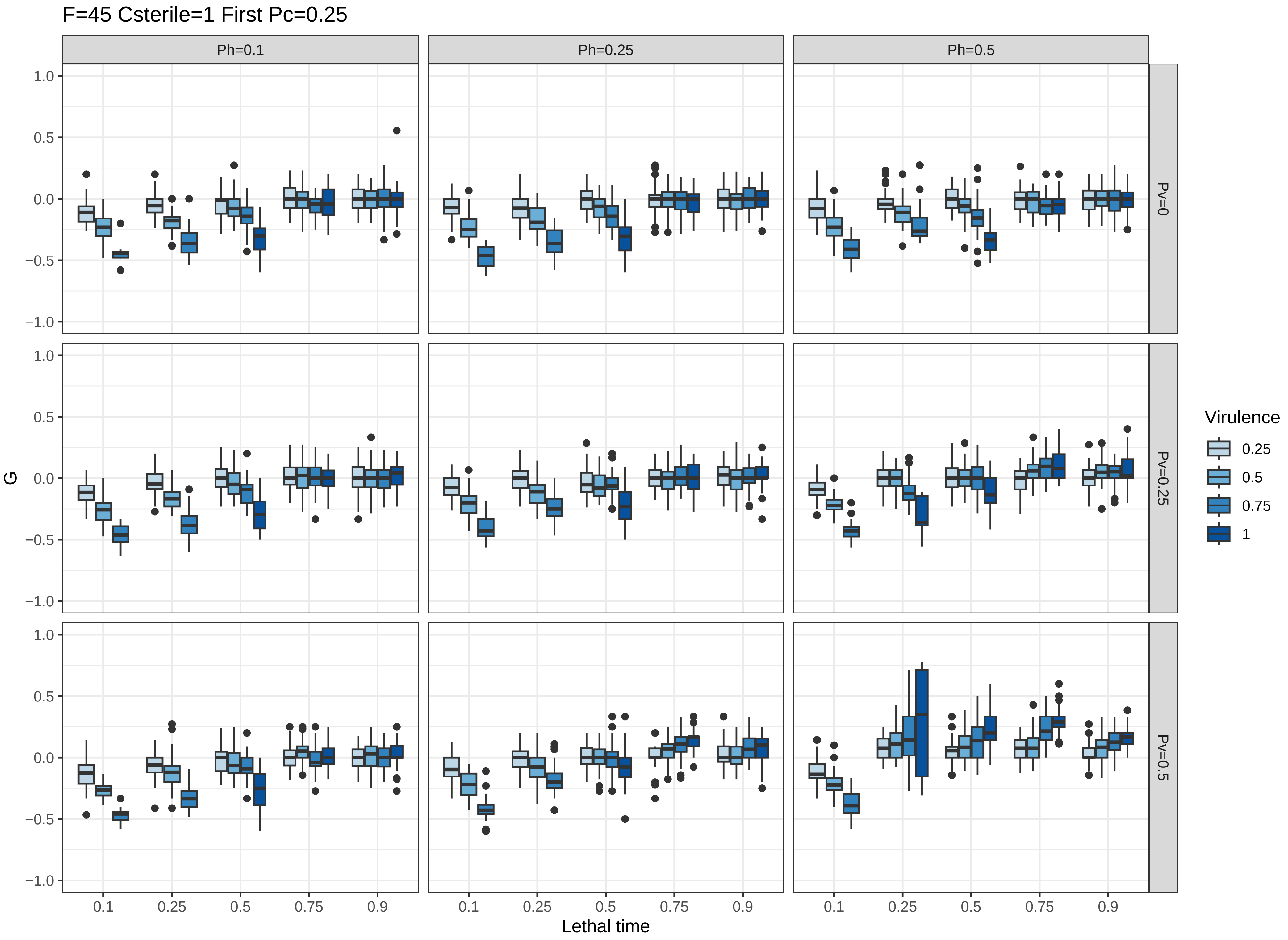

F=45 Csterile=1 First Pc=0.25
Ph=0.1
Ph=0.25
Ph=0.5
Pv=0
Pv=0.25
Pv=0.5
G
1.0
0.5
0.0
−0.5
−1.0
0.1
0.25
0.5
0.75
0.9
Lethal time
Virulence
0.25
0.5
0.75
1

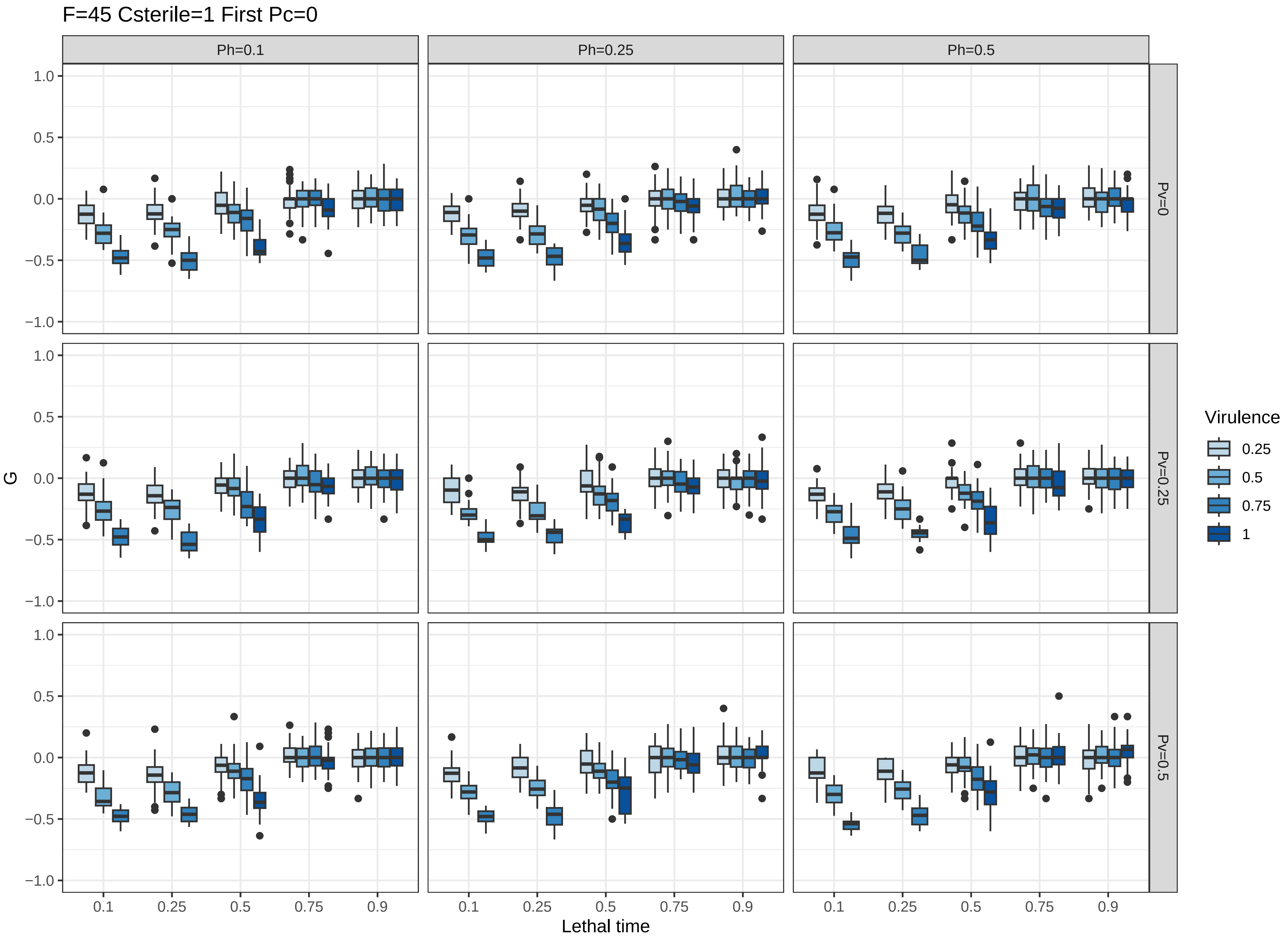

F=45 Csterile=1 First Pc=0
Ph=0.1
Ph=0.25
Ph=0.5
Pv=0
Pv=0.25
Pv=0.5
G
1.0
0.5
0.0
−0.5
−1.0
0.1
0.25
0.5
0.75
0.9
Lethal time
Virulence
0.25
0.5
0.75
1

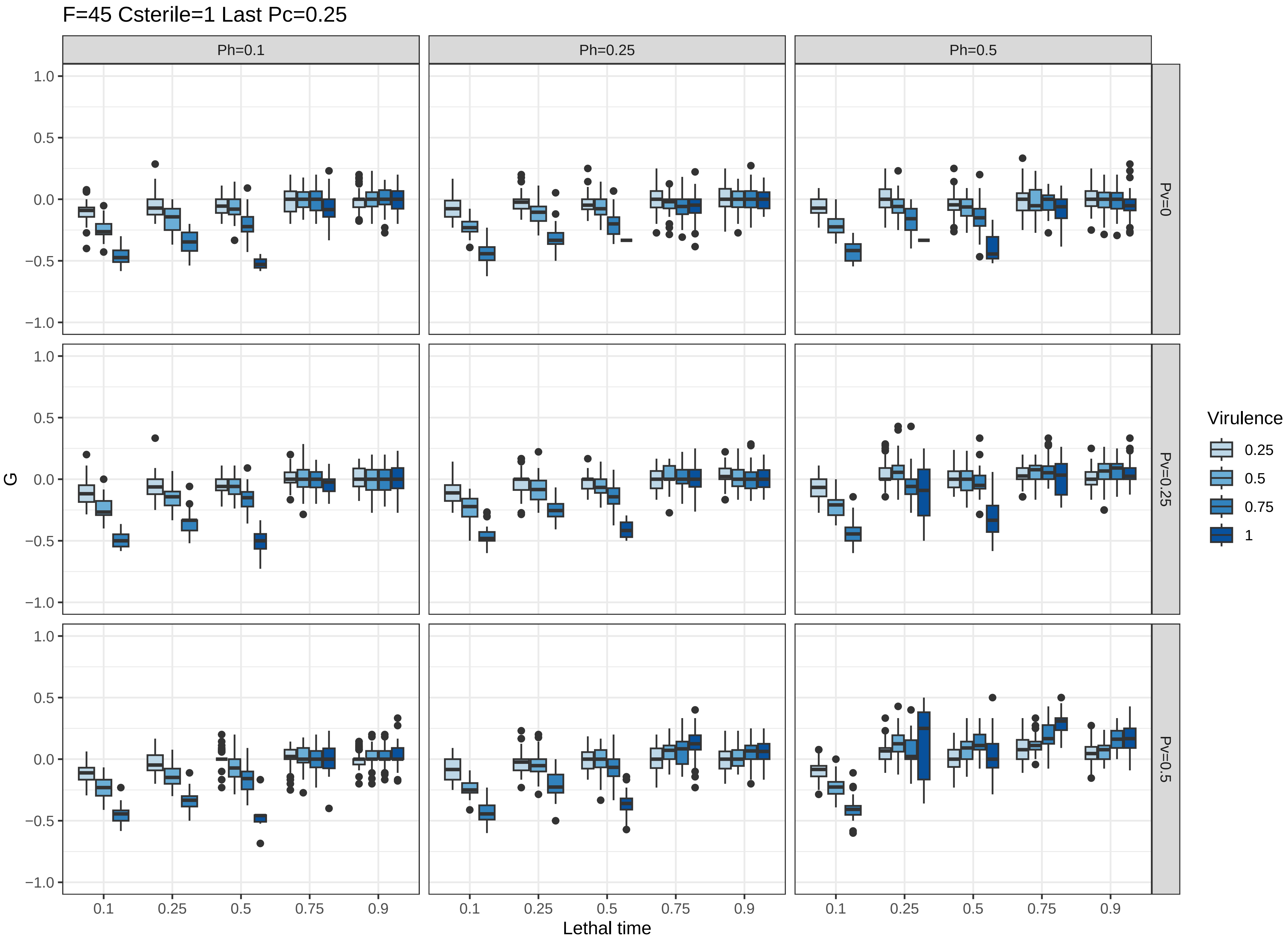

F=45 Csterile=1 Last Pc=0.25
Ph=0.1
Ph=0.25
Ph=0.5
Pv=0
Pv=0.25
Pv=0.5
G
Lethal time
Virulence
0.25
0.5
0.75
1

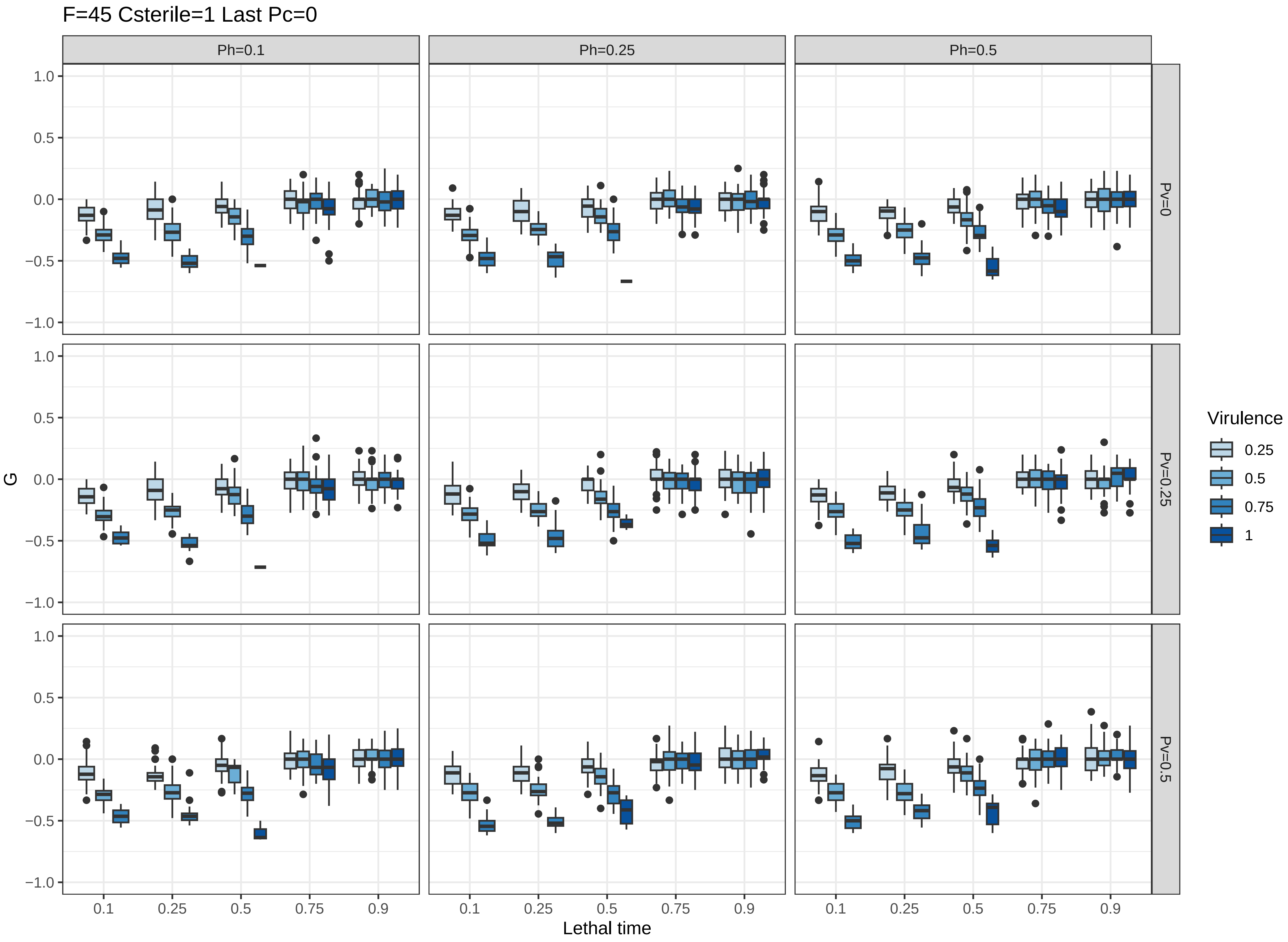

F=45 Csterile=1 Last Pc=0
Ph=0.1
Ph=0.25
Ph=0.5
Pv=0
Pv=0.25
Pv=0.5
G
1.0
0.5
0.0
−0.5
−1.0
0.1
0.25
0.5
0.75
0.9
Lethal time
Virulence
0.25
0.5
0.75
1

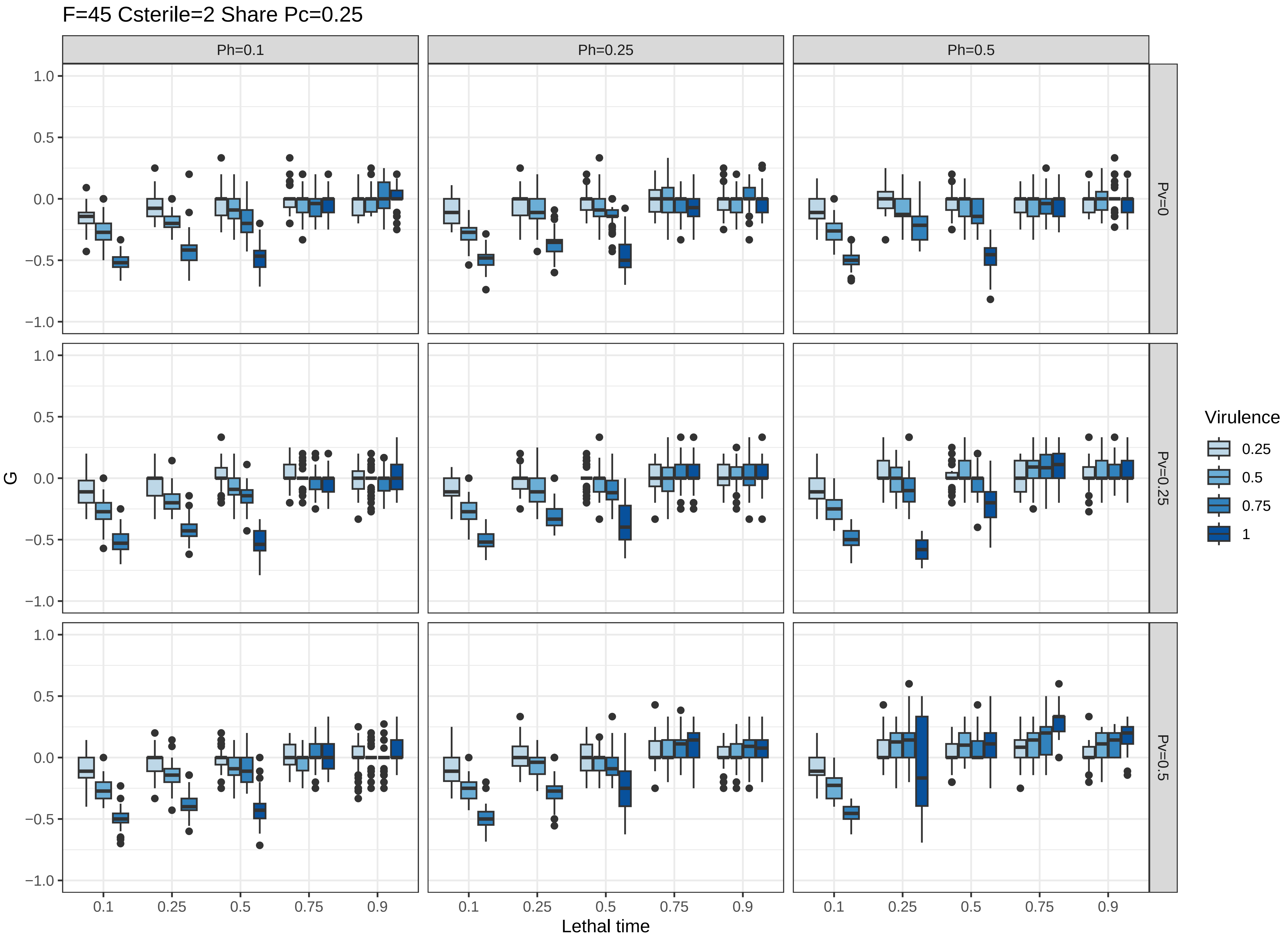

F=45 Csterile=2 Share Pc=0.25
Ph=0.1
Ph=0.25
Ph=0.5
Pv=0
Pv=0.25
Pv=0.5
G
Lethal time
Virulence
0.25
0.5
0.75
1
1.0
0.5
0.0
−0.5
−1.0
0.1
0.25
0.5
0.75
0.9

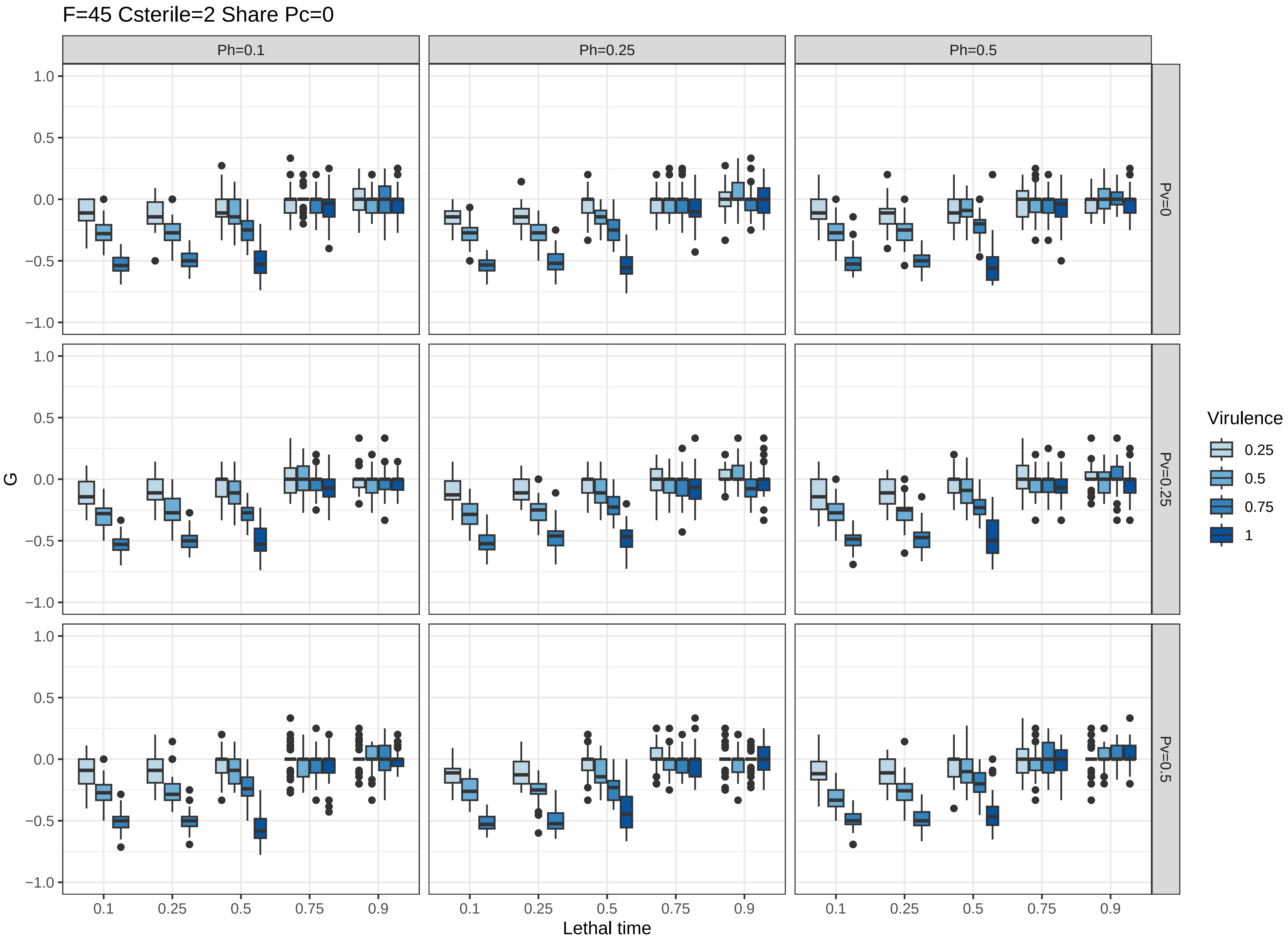

F=45 Csterile=2 Share Pc=0
Ph=0.1
Ph=0.25
Ph=0.5
Pv=0
Pv=0.25
Pv=0.5
1.0
0.5
0.0
−0.5
−1.0
G
0.1
0.25
0.5
0.75
0.9
Lethal time
Virulence
0.25
0.5
0.75
1

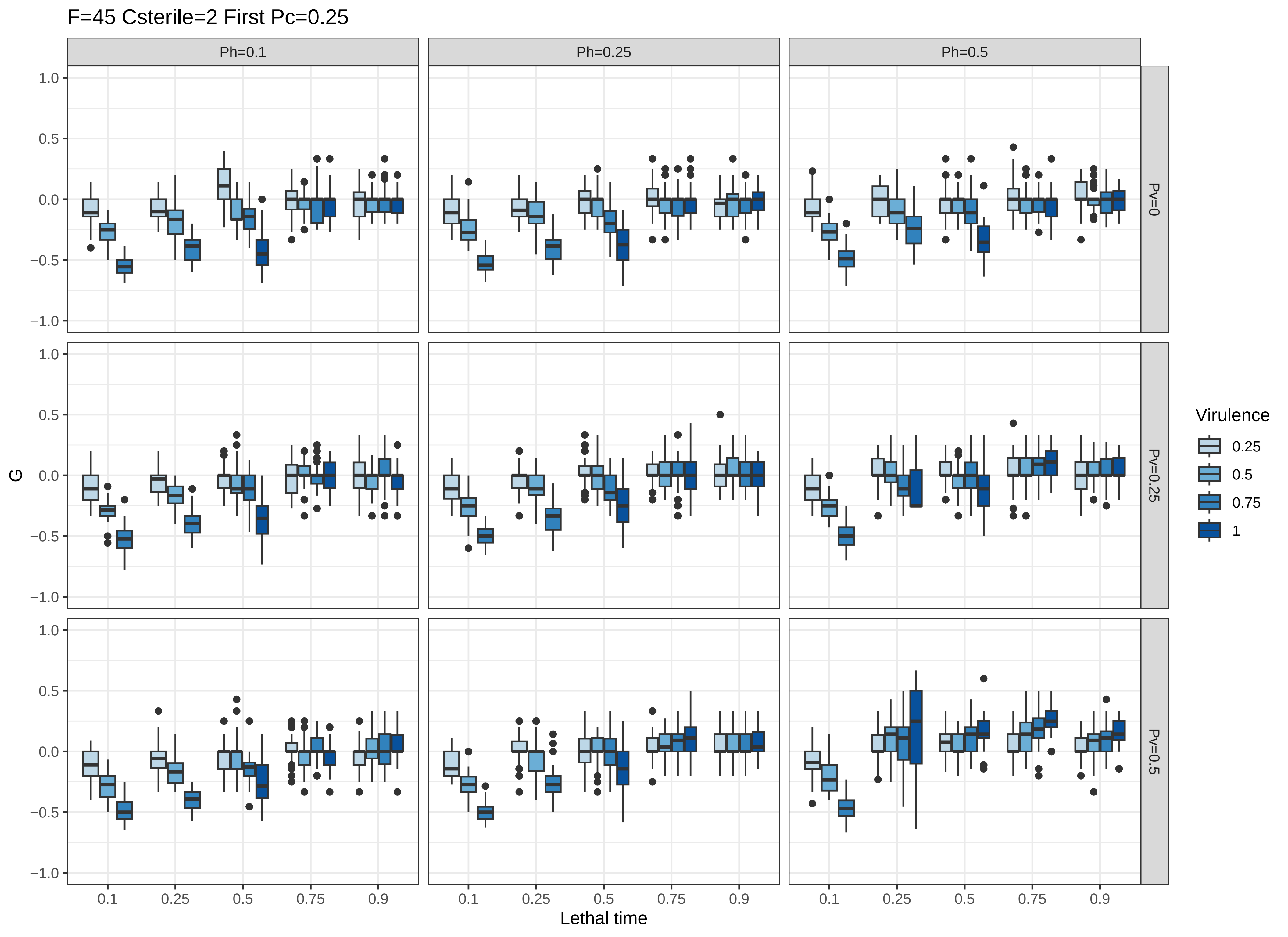

F=45 Csterile=2 First Pc=0.25
Ph=0.1
Ph=0.25
Ph=0.5
Pv=0
Pv=0.25
Pv=0.5
G
1.0
0.5
0.0
−0.5
−1.0
0.1
0.25
0.5
0.75
0.9
Lethal time
Virulence
0.25
0.5
0.75
1

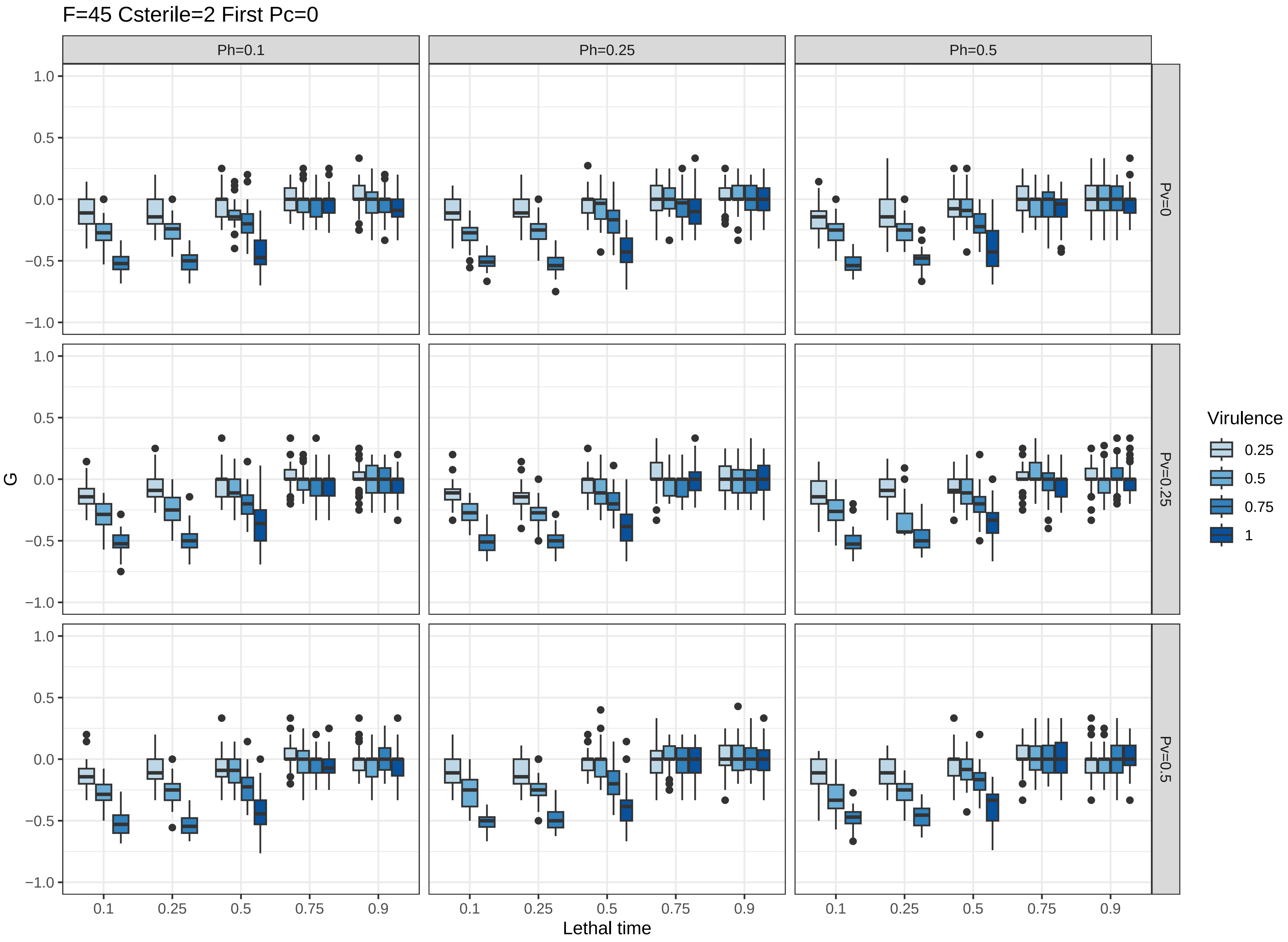

F=45 Csterile=2 First Pc=0
Ph=0.1
Ph=0.25
Ph=0.5
Pv=0
Pv=0.25
Pv=0.5
G
Lethal time
Virulence
0.25
0.5
0.75
1

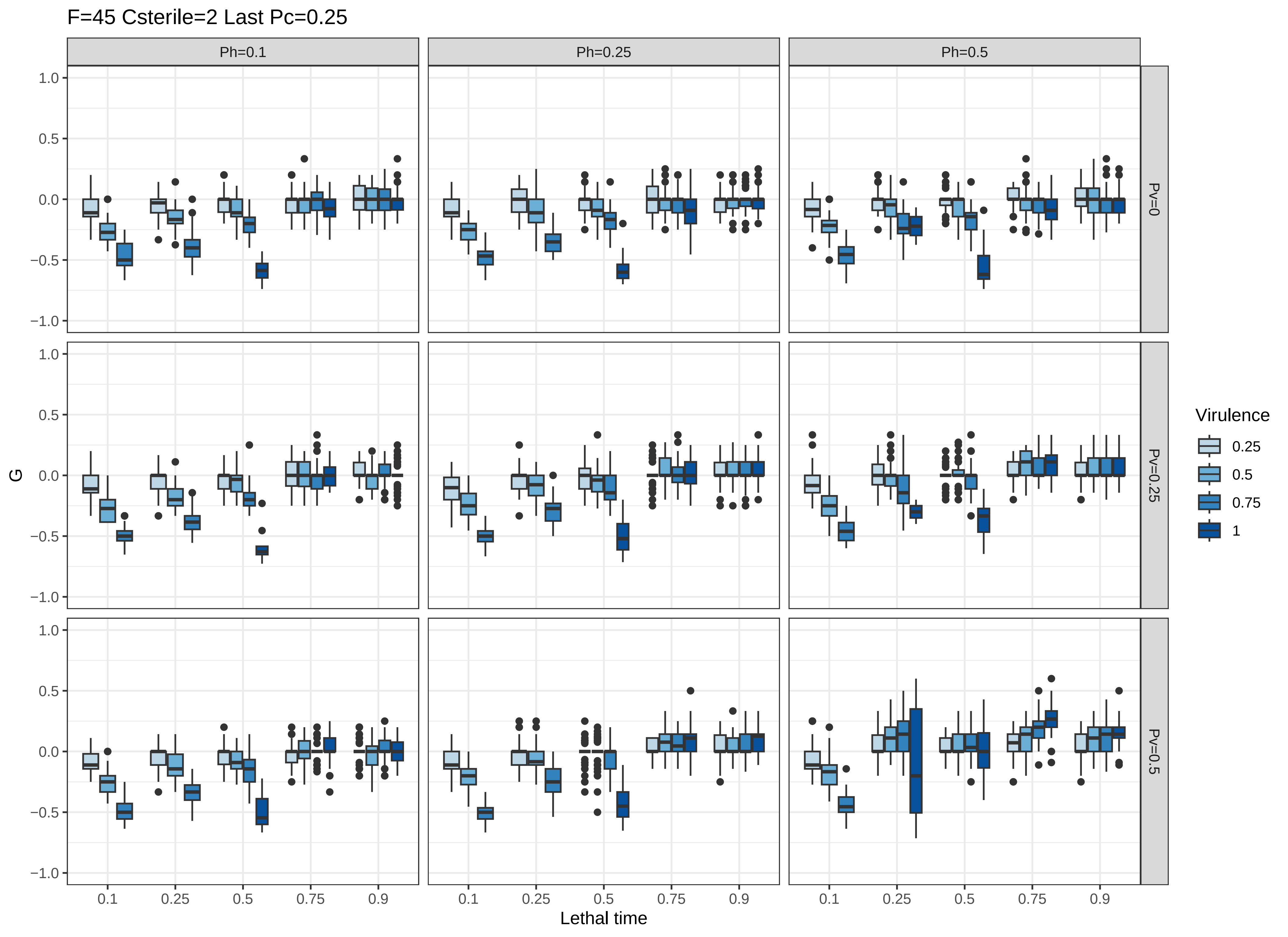

F=45 Csterile=2 Last Pc=0.25
Ph=0.1
Ph=0.25
Ph=0.5
Pv=0
Pv=0.25
Pv=0.5
1.0
0.5
0.0
−0.5
−1.0
G
0.1
0.25
0.5
0.75
0.9
Lethal time
Virulence
0.25
0.5
0.75
1

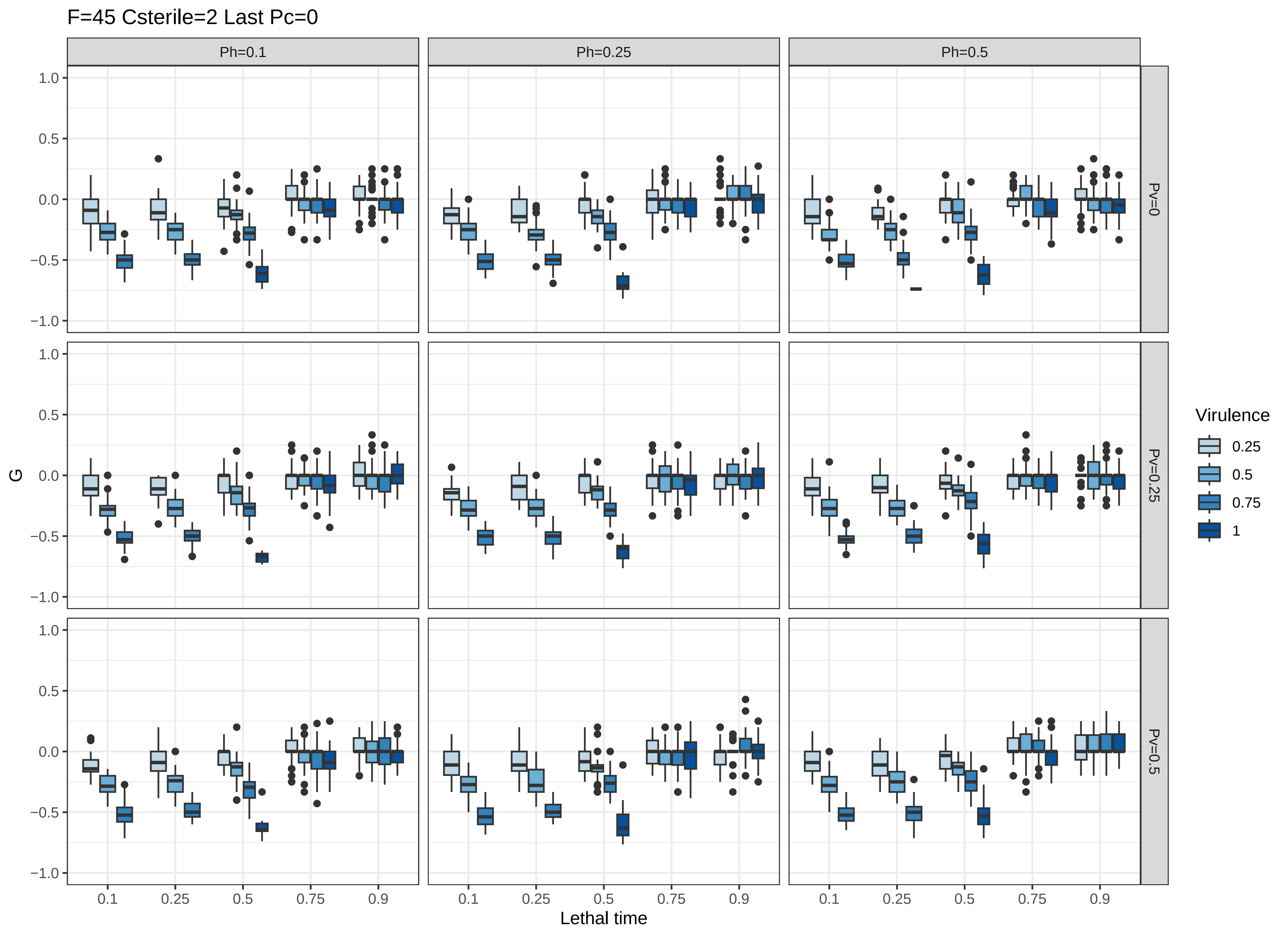

F=45 Csterile=2 Last Pc=0
Ph=0.1
Ph=0.25
Ph=0.5
Pv=0
Pv=0.25
Pv=0.5
1.0
0.5
0.0
−0.5
−1.0
G
0.1
0.25
0.5
0.75
0.9
Lethal time
Virulence
0.25
0.5
0.75
1
