# Supplementary material for: Life history traits of the target pest and transmission routes of the biocide are critical for the success of the boosted Sterile Insect Technique
Source: Curr Res Insect Sci. 2024 Nov 12;6:100101. doi: 10.1016/j.cris.2024.100101 (PMC11612786; doi:10.1016/j.cris.2024.100101)
Supplement: Supplementary file 3 [file mmc3.docx]

# Supplementary materials

**S1**: Illustration of the conditions for stopping the simulations for the three scenarios: (1) No control treatment, (2) control with SIT and (3) control with boosted SIT. Ratio_SIT and ratio_SIT_b are the ratio of released sterile males/wild males for SIT and boosted SIT respectively. In green we show examples of values that would stop the simulations with the success of control techniques.

**S2**: Ratio of boosted SIT success compared to SIT (*G*) in relation to the biocide's lethal time (*0.01, 0.25, 0.5, 0.75, 0.9*), virulence (*blue levels: 0.25, 0.5, 0.75, 1*), the transmission rate during mating (*Ph: 0.1, 0.25, 0.5*) and to offspring (*Pv*: *0, 0.5,0.75*), the fecundity of females (*F: 6, 45*), the mating competitiveness of sterile males (*Csterile: 0.5, 1, 2*), the spermatic competition (*Csperm: “first”, ”share”, “last”*), and the transmission rate of the biocides during leks (*Pc: 0.25, 0*).
